# Prognosis of cirrhotic patients admitted to intensive care unit: a meta-analysis

**DOI:** 10.1186/s13613-017-0249-6

**Published:** 2017-03-21

**Authors:** Delphine Weil, Eric Levesque, Marc McPhail, Rodrigo Cavallazzi, Eleni Theocharidou, Evangelos Cholongitas, Arnaud Galbois, Heng Chih Pan, Constantine J. Karvellas, Bertrand Sauneuf, René Robert, Jérome Fichet, Gaël Piton, Thierry Thevenot, Gilles Capellier, Vincent Di Martino, METAREACIR Group, Vincent Di Martino, Delphine Weil, Thierry Thévenot, Jean-Paul Cervoni, Carine Richou, Claire Vanlemmens, Stavros Dritsas, Gilles Capellier, Gaël Piton, Claire Chaignat, Sébastien Pili-Floury, Gilles Blasco, Emmanuel Samain, Didier Samuel, Eric Levesque, Faouzi Saliba, Philippe Ichaï, Arnaud Galbois, Bertrand Guidet, Vincent Das, Bertrand Sauneuf, Jean-Paul Mira, Dominique Perrotin, Benoit Champigneulle, Frédéric Pene, René Robert, Bruno Filloux, Christine Silvain, Jérome Fichet, Marc McPhail, Julia Wendon, Debbie Shawcross, William Bernal, Eleni Theocharidou, Banwari Agarwal, Andrew K. Burroughs†, Evangelos Cholongitas, Rodrigo Cavallazzi, Constantine J. Karvellas, Heng Chih Pan, Yung-Chang Chen, Ming-Hung Tsai

**Affiliations:** 1 Hepatology Department, University Hospital Jean Minjoz, 3 bld Fleming, 25030 Besançon, France; 2 Centre Hépato-Biliaire, University Hospital Paul Brousse, Villejuif, France; 3 Liver Intensive Care Unit and Institute of Liver Studies and Transplantation, King’s College Hospital, London, UK; 4 Intensive Care Unit, University-Louisville, Louisville, KY USA; 5 Royal Free Sheila Sherlock Liver Centre, Royal Free Hospital, London, UK; 6 Liver Department, Aristotle University of Thessaloniki, Thessaloníki, Greece; 7 Intensive Care Unit, University Hospital Saint-Antoine, Paris, France; 8 Nephrology Department, Chang Gung Memorial Hospital, Taipei, Taiwan; 9 Hepatology Department, Intensive Care Unit, University of Alberta, Edmonton, Canada; 10 Intensive Care Unit, Hôpital Cochin, Paris, France; 11 Intensive Care Unit, University Hospital of Poitiers, Poitiers, France; 12 Intensive Care Unit, University Hospital of Tours, Tours, France; 13 Intensive Care Unit, University Hospital Jean Minjoz, Besançon, France

**Keywords:** Cirrhosis, Extrahepatic organ failure, Organ replacement therapy, Mortality, Prognostic scores, CLIF-SOFA, MELD

## Abstract

**Background:**

The best predictors of short- and medium-term mortality of cirrhotic patients receiving intensive care support are unknown.

**Methods:**

We conducted meta-analyses from 13 studies (2523 cirrhotics) after selection of original articles and response to a standardized questionnaire by the corresponding authors. End-points were in-ICU, in-hospital, and 6-month mortality in ICU survivors. A total of 301 pooled analyses, including 95 analyses restricted to 6-month mortality among ICU survivors, were conducted considering 249 variables (including reason for admission, organ replacement therapy, and composite prognostic scores).

**Results:**

In-ICU, in-hospital, and 6-month mortality was 42.7, 54.1, and 75.1%, respectively. Forty-eight patients (3.8%) underwent liver transplantation during follow-up. In-ICU mortality was lower in patients admitted for variceal bleeding (OR 0.46; 95% CI 0.36–0.59; *p* < 0.001) and higher in patients with SOFA > 19 at baseline (OR 8.54; 95% CI 2.09–34.91; *p* < 0.001; PPV = 0.93). High SOFA no longer predicted mortality at 6 months in ICU survivors. Twelve variables related to infection were predictors of in-ICU mortality, including SIRS (OR 2.44; 95% CI 1.64–3.65; *p* < 0.001; PPV = 0.57), pneumonia (OR 2.18; 95% CI 1.47–3.22; *p* < 0.001; PPV = 0.69), sepsis-associated refractory oliguria (OR 10.61; 95% CI 4.07–27.63; *p* < 0.001; PPV = 0.76), and fungal infection (OR 4.38; 95% CI 1.11–17.24; *p* < 0.001; PPV = 0.85). Among therapeutics, only dopamine (OR 5.57; 95% CI 3.02–10.27; *p* < 0.001; PPV = 0.68), dobutamine (OR 8.92; 95% CI 3.32–23.96; *p* < 0.001; PPV = 0.86), epinephrine (OR 5.03; 95% CI 2.68–9.42; *p* < 0.001; PPV = 0.77), and MARS (OR 2.07; 95% CI 1.22–3.53; *p* = 0.007; PPV = 0.58) were associated with in-ICU mortality without heterogeneity. In ICU survivors, eight markers of liver and renal failure predicted 6-month mortality, including Child–Pugh stage C (OR 2.43; 95% CI 1.44–4.10; *p* < 0.001; PPV = 0.57), baseline MELD > 26 (OR 3.97; 95% CI 1.92–8.22; *p* < 0.0001; PPV = 0.75), and hepatorenal syndrome (OR 4.67; 95% CI 1.24–17.64; *p* = 0.022; PPV = 0.88).

**Conclusions:**

Prognosis of cirrhotic patients admitted to ICU is poor since only a minority undergo liver transplant. The prognostic performance of general ICU scores decreases over time, unlike the Child–Pugh and MELD scores, even recorded in the context of organ failure. Infection-related parameters had a short-term impact, whereas liver and renal failure had a sustained impact on mortality.

**Electronic supplementary material:**

The online version of this article (doi:10.1186/s13613-017-0249-6) contains supplementary material, which is available to authorized users.

## Background

The natural course of liver cirrhosis is often punctuated by life-threatening complications requiring admission to an intensive care unit (ICU) [1]. Critically ill cirrhotic patients were reported to have poor prognosis [2–4], but recent data suggest improvements due to significant progress in the management of general in-ICU populations, and a better understanding of the pathophysiology of cirrhosis [5–7]. The generalization of standardized interventions, such as management of portal hypertension-related bleeding, has contributed to a survival gain in this population. However, survival after hospital discharge remains low in cirrhotic patients [3, 8–10]. Numerous studies have focused on prognostic factors of cirrhotic patients admitted in ICU but provided short-term and controversial results, possibly impacted by selection biases. In the more recent studies on the topic, the performances of some isolated characteristics such as acute kidney injury [11–14], need for mechanical ventilation [3, 15, 16], septic shock [5, 17], and of liver-specific or general ICU scores [2, 7, 18–21] to predict mortality have been compared but the design was often retrospective and reflected a single-center experience. As a consequence, no evidence-based recommendation regarding the admission of critically ill cirrhotic patients to ICUs can be made [22]. More importantly, the long-term prognosis of the patients who survived to intensive care and its determinants has been poorly documented.

The aim of this meta-analysis was therefore to compare the prognostic value of a large set of variables on in-ICU, in-hospital, and 6-month mortality in patients who survived the ICU phase.

## Methods

### Literature search (Additional file 1: Figure S1)

Computer searches for eligible studies were performed in April 2014 using PubMed, Cochrane Library, and EMBASE, with the following combination of key words: «cirrhosis» OR «cirrhotic» AND «intensive care» OR «ICU» OR «critically ill» in the title AND «prognosis» OR «survival» OR «outcome» OR «mortality» in the title or abstract, with a publication date ranging from January 2004 to March 2014. Reference lists of identified articles were also manually searched for additional relevant publications. Two main authors (DW and VDM) selected independently studies published as full papers in English, and in the event of divergence on studies’ selection, a consensus was reached through discussion. The study design could be prospective or retrospective, but the main outcome measure of the study had to be survival.

### Data collection

Because the published studies were not fully comparable, a standardized questionnaire was sent to the principal investigators of eligible studies asking them to extract from their database the distribution of survivors and non-survivors at each time point (in ICU, in hospital, at 6 months after discharge from ICU) for 249 variables. To avoid any heterogeneity in the answers, qualitative variables were defined if necessary, and units of each quantitative variable were given in the questionnaire. Quantitative variables were presented as intervals or cutoffs, according to their clinical relevance. The queries were focused on (1) characteristics of the center (primary/tertiary, having or not a liver transplantation program, a dedicated Hepatology department, TIPS being or not routinely performed) and of the ICU (general or specialized for liver diseases, involved or not in the postoperative follow-up after liver transplantation, strategies for limitation of life-sustaining treatments being available or not), number of ICU admissions per year, and proportion of cirrhotic patients; (2) period of inclusion; (3) baseline patient characteristics (age, sex, history of cirrhosis, main reason for admission in ICU, clinical and biological characteristics); (4) biological and clinical characteristics during ICU stay; (5) therapeutics used during ICU stay (required or not on admission); (6) prognostic scores including Child–Pugh and MELD scores, SOFA and its variation between baseline and day 3, modified SOFA (mSOFA), CLIF-SOFA, APACHE-II, and number of non-hematologic organ failures.

### Statistical analyses

For each variable, pooled analyses comparing survivors and non-survivors were performed. We used the DerSimonian and Laird model for random effects to obtain summary estimates across studies. Results were expressed by the combined weight-adjusted odds ratios (OR), with their 95% confidence interval (95% CI). A *p* value (from directional zero-effect test) of 0.05 or less was considered statistically significant. We tested for heterogeneity using Cochran’s *Q* test (considered significant at *p* ≤ 0.05). Positive and negative predictive values were calculated for significant results. This report follows the MOOSE guidelines for reporting meta-analyses of observational studies [23].

## Results

### Study characteristics

We selected 30 studies published between January 01, 2004, and March 31, 2014, including 6030 patients. Nine authors (ten studies) could not be contacted [15, 24–27] or did not answer [16, 20, 28–30]; three authors (five studies) agreed to participate but did not return the questionnaire [13, 17, 31–33]; two authors had no longer access to the database [9, 34]. Finally, we received 11 answers, covering 13 studies (2523 patients, Table 1; Additional file 1: Figure S1) [2, 3, 5–8, 10, 35–40]. Studies from Jenq et al. [37, 38] and from Levesque et al. [2, 3] were grouped for avoiding double-counting patients in the meta-analyses.

**Table 1:** Studies included in the meta-analysis and major outcome events

Study	Number of cirrhotics	Period of inclusion	Mortality in ICU *N* (%)	Mortality in hospital *N* (%)	Mortality at 3 months *N* (%)	Mortality at 6 months *N* (%)	Transplanted at 3 months *N* (%)/6 months *N* (%)	Study design	Specificity of studied population
Jenq [37, 38]	188	2005–2006	95 (50.5)	137 (72.8)	154 (81.9)	157 (83.5)	0 (0.0)/0 (0.0)	Prospective	
Fichet [10]	71	1995–2005	25 (35.2)	28 (39.4)	NA	NA	0 (0.0)/0 (0.0)	Retrospective	100% stage III/IV HE^a^
Filloux [36]	83	2002–2006	29 (34.9)	39 (46.9)	NA	47 (56.6)	NA	Retrospective	
Karvellas [39]	178	2003–2005	NA	91 (51.1)	NA	NA	NA	Retrospective	
Das [8]	138	2005–2008	57 (41.3)	75 (54.3)	NA	NA	NA	Retrospective	
Galbois [6]	56	1995–1998	22 (39.2)	25 (44.6)	NA	NA	NA	Retrospective	
Levesque [2, 3]	451	2005–2011	17 (38.8)	208 (46.2)	189 (76.8)	211 (85.7)	27 (5.9)/28 (6.2)	(2) Prospective/(3) retrospective	(2) 0% PO (3) 100% IMV
Cavallazzi [35]	441	2003–2009	NA	213 (48.3)	NA	NA	15 (3.4)/15 (3.4)	Retrospective	
Shawcross [40]	563	2000–2007	256 (45.5)	334 (59.3)	NA	NA	NA	Prospective	
Sauneuf [5]	89	1997–2010	64 (71.9)	74 (83.1)	77 (86.5)	77 (86.5)	0 (0.0)/2 (2.2)	Retrospective	100% septic shock
Theocharidou [7]	265	2005–2012	91 (34.3)	125 (47.1)	168 (63.3)	173 (65.2)	NA	Retrospective	

*NA* not available, *HE* hepatic encephalopathy, *PO* postoperative, *IMV* invasive mechanical ventilation

^a^According to West Haven classification

### Center characteristics

The 13 studies came from ten tertiary centers, of which eight had a liver transplantation program and nine had a hepatology department. In seven centers, TIPS was routinely performed. Of the ten ICUs, seven were generalized ICUs and nine had strategies for limitation of life-sustaining therapies available. The total number of ICU admissions per year was >1000 in four centers and <500 in one center. The reported proportion of cirrhotic patients was <5% in three centers, 5–10% in one, 10–20% in one, and >20% in three centers.

### Data recorded

In-ICU mortality was reported in ten studies (1904 patients), in-hospital mortality in 12 studies (2446 patients), and 6-month mortality in five studies (828 patients).

Of the 249 listed variables, 92 were available in none or only one questionnaire and thus could not be further analyzed. The majority of them were related to events occurring during ICU stay.

### Study population and major outcome events

The patients’ characteristics on ICU admission are detailed in Additional file 1: Table S1. Briefly, two-third of patients were males and <60 years; cirrhosis was alcohol-related in 58.4% and associated with hepatocellular carcinoma in 11.7% of cases; the main primary reason for admission was variceal bleeding.

**Fig. 1:**
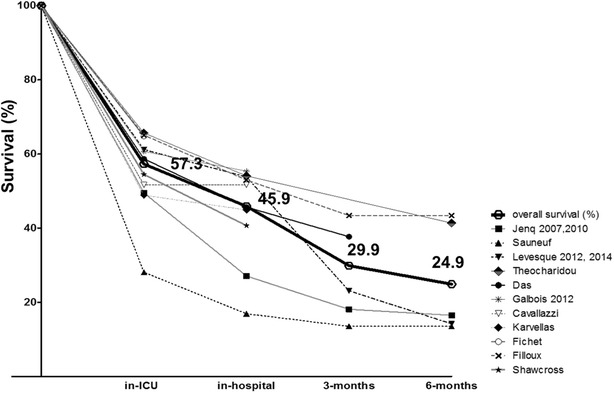
Overall survival. In-ICU, in-hospital, 3-month, and 6-month survival rates are reported for each study included (*thin* and *dotted lines*) and for the whole study population (*heavy black line*). On the *x*-axis, the timescale is not complied, given the variable length of stay in ICU and hospital

Overall, 814 patients (42.7%) died in the ICU, 1322 (54.1%) died in hospital, and 622 (75.1%) had died at 6 months. Among the studies, in-ICU, in-hospital, and 6-month mortality ranged from 34.3 to 71.9, 39.4 to 83.1, and 56.6 to 86.5%, respectively (Fig. 1). Among the 1240 patients with available information, only 48 (3.8%) underwent liver transplantation within the first 6 months of follow-up. The majority (45 patients) were transplanted within the first 3 months. The reasons why patients did or did not undergo liver transplantation were not given.

### Predictors of short-term mortality

**Fig. 2:**
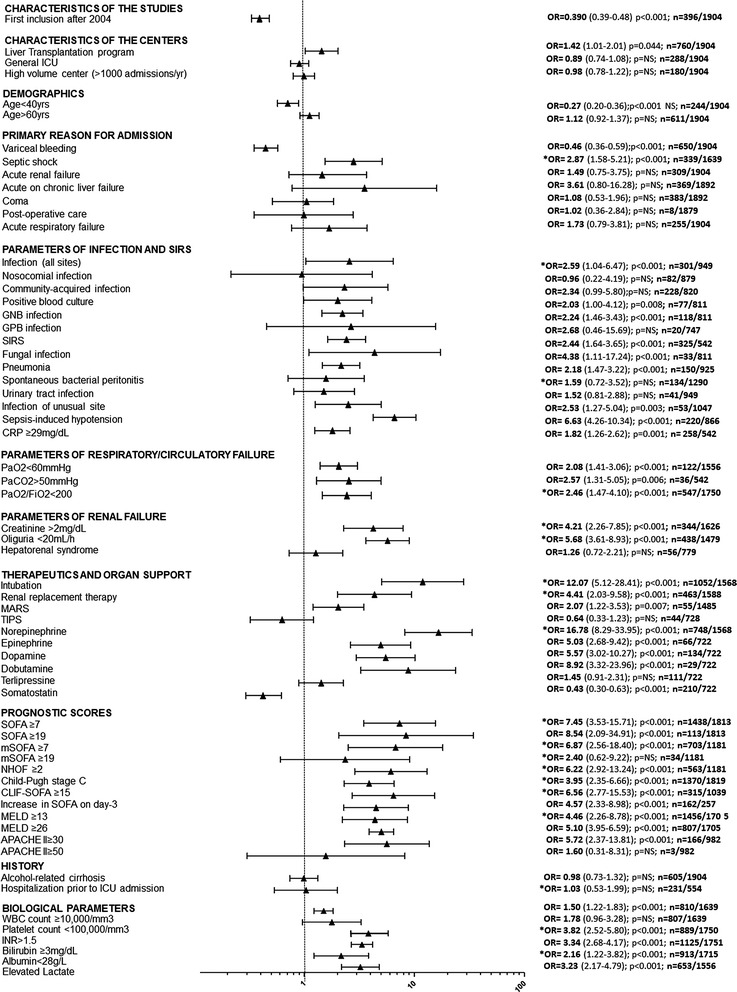
Predictors of in-ICU mortality. For each variable, combined weight-adjusted odds ratios (*filled triangle*) and their 95% confidence intervals (*horizontal line*) obtained from forest plots of pooled analyses are reported, together with the *p* value, the number of cirrhotic patients with variable present, and the total number of patients with available data. Odds ratios >1 correspond to variables associated with higher mortality. Odds ratios <1 correspond to variables associated with better survival. Odds ratios with a 95% CI containing 1 correspond to nonsignificant results. (*) indicates heterogeneous results. *APACHE* Acute Physiology and Chronic Health Evaluation, *CRP* C-reactive protein, *GNB* Gram-negative Bacilli, *GPB* Gram-positive Bacilli, *MARS* molecular adsorbents recirculation system, *MELD* model of end-stage liver disease, *NHOF* non-hematologic organ failure, *NS* not significant, *SIRS* systemic inflammatory response syndrome, *TIPS* transjugular intrahepatic portosystemic shunt, *SOFA* Sequential Organ Failure Assessment, *mSOFA* modified SOFA, *CLIF-SOFA* modified SOFA according to the Chronic Liver Failure Consortium of the European Association for the Study of the Liver

For each meta-analysis, Additional file 1: Tables S2 and S3 show the weight-adjusted combined OR, its 95% CI, and the positive and negative predictive values for mortality. A selection of the more relevant predictors of in-ICU mortality is given in Fig. 2.

#### Center characteristics

Centers with a liver transplantation program were associated with higher in-ICU mortality. Centers without routine TIPS programs had higher in-hospital mortality. Conversely, in-ICU and in-hospital mortality was not different in centers having a dedicated liver department or not, in ICUs involved in post-transplantation care or not, in centers with or without available strategies for limitation of life-sustaining therapies, in high-volume or low-volume centers regarding the total number of ICU admissions or the proportion of cirrhotic patients admitted to ICU per year.

#### Period of inclusion

The inclusion period ranged from 1995 to 2012. In-ICU mortality was higher in studies with inclusions before the year 2004 [5, 6, 10] versus others. The same results were observed for in-hospital mortality.

#### Demographics and history of cirrhosis

**Fig. 3:**
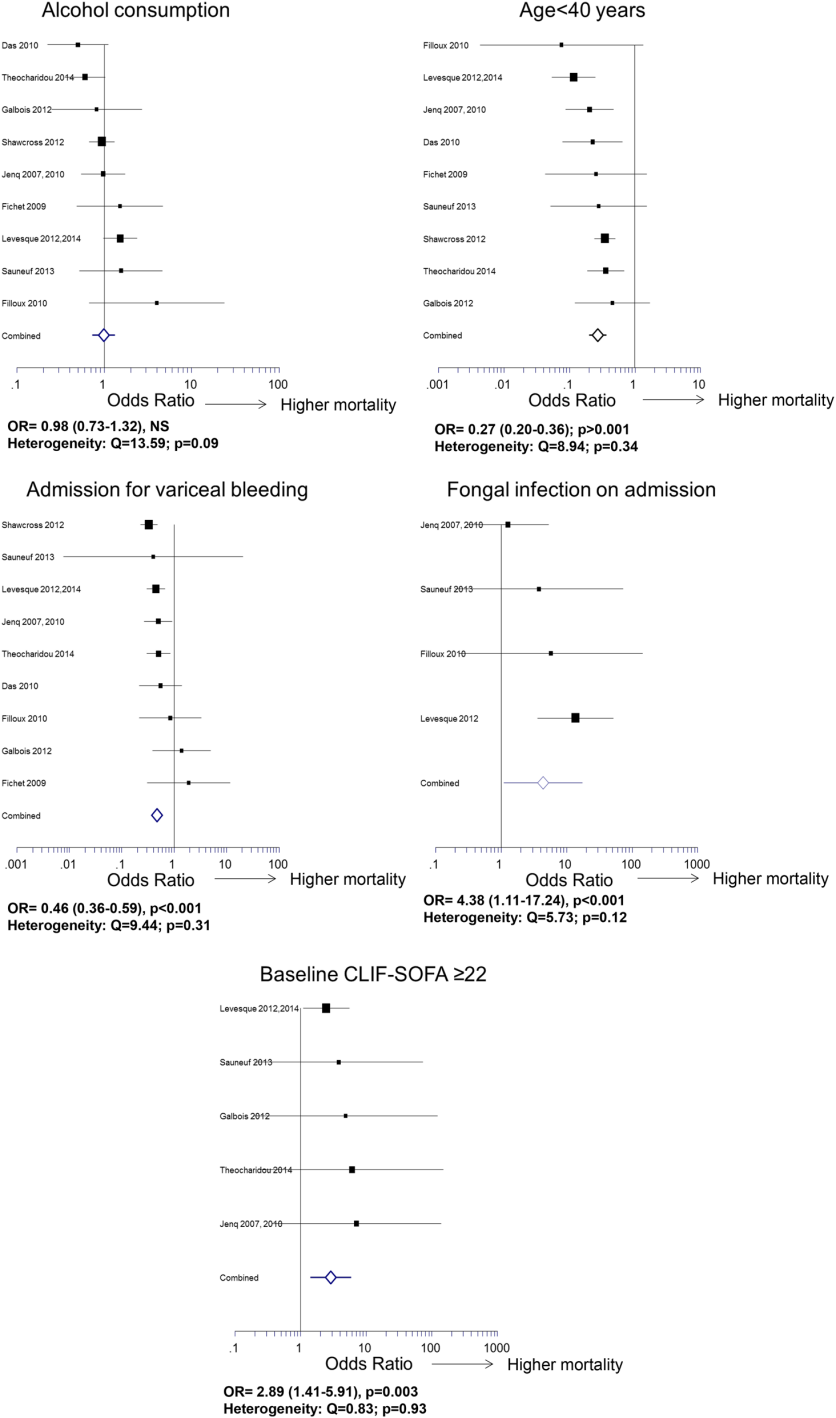
Some remarkable results regarding in-ICU mortality (forest plots of odds ratios)

Age <40 years was associated with lower in-ICU (Figs. 2 and 3) and in-hospital mortality. Age >60 years was associated with higher in-hospital mortality, but results were not significant for in-ICU mortality. Sex had no influence on short-term mortality. The outcome of patients with alcohol-induced cirrhosis did not differ from that of patients with other causes (Figs. 2 and 3).

#### Circumstances of admission

Mortality was not different in patients directly admitted to ICUs as compared to patients who were previously hospitalized. Among the reasons for admission, variceal bleeding was associated with lower in-ICU mortality (Fig. 3), whereas septic shock was associated with poor outcome, but with heterogeneous results (Additional file 1: Table S2 and Fig. 2).

#### SIRS and sepsis

SIRS was associated with higher in-ICU (Fig. 2) and in-hospital mortality. Baseline CRP levels >29 mg/L were associated with higher in-ICU mortality. Sepsis-induced hypotension was associated with higher in-ICU and in-hospital mortality. In septic patients, refractory oliguria was a strong predictor of in-ICU and in-hospital mortality. Nosocomial and community-acquired infections had similar outcomes (Fig. 2). Patients with positive blood culture, pneumonia, or infection of unusual site were at higher risk of death in-ICU. Spontaneous bacterial peritonitis only impacted on in-hospital mortality. Gram-negative bacillus infections were associated with higher in-ICU mortality. Fungal infection was associated with higher in-ICU (Figs. 2 and 3) and in-hospital mortality.

#### Respiratory function and hemodynamics

A PaO_2_ < 60 mmHg at baseline was associated with higher in-ICU and in-hospital mortality. A PaCO_2_ > 50 mmHg was associated with higher in-ICU mortality. A pH <7.3 was associated with higher in-hospital mortality, but results were heterogeneous for in-ICU mortality. A PaO_2_/FiO_2_ ratio <100 was associated with higher in-ICU mortality. A baseline mean arterial pressure <65 mmHg was associated with poor short-term outcome, but pooled analyses provided heterogeneous results. High lactate levels at baseline were associated with higher in-ICU and in-hospital mortality.

#### Renal function

Four markers of renal function at baseline were reported for all studies. Hepatorenal syndrome did not impact on in-ICU or in-hospital mortality. A serum creatinine level >2 mg/dL was associated with higher in-hospital and in-ICU mortality, but results were heterogeneous. Results were also heterogeneous for serum creatinine >1.5 mg/dL and oliguria, defined as urine output ≤20 mL/h.

#### Neurological status

Among the five available markers of neurological status, none had significant impact on mortality without heterogeneity.

#### Medical interventions and organ replacement therapies

Investigators were queried about 15 medical interventions, including nine drugs and three organ replacement therapies. Of these, only ten interventions could be studied. Answers were insufficient for nutritional support and the use of insulin, hydrocortisone, and *N*-acetylcysteine. The most relevant information for prognosis was given by the use of epinephrine, which was associated with higher in-ICU and in-hospital mortality. The use of dopamine and dobutamine was associated with higher in-ICU mortality. The use of norepinephrine, regardless of its indication (hemodynamic support or hepatorenal syndrome), provided heterogeneous results in terms of in-ICU mortality. The use of somatostatin was associated with lower in-ICU mortality. Among organ replacement therapies, MARS was associated with in-ICU and in-hospital mortality. Mechanical ventilation and renal replacement therapy were also associated with poorer short-term outcomes, but the results were heterogeneous (Fig. 2).

#### Prognostic scores

Twenty-four analyses regarding the relevance of prognostic scores for predicting mortality at each given time were performed. The number of organ failures strongly impacted on in-ICU mortality. A SOFA > 19, a CLIF-SOFA ≥ 22 (Fig. 3), and an APACHE-II ≥ 30 were strongly associated with higher in-ICU mortality, whereas values of the mSOFA provided heterogeneous results (Figs. 2 and 3). Increased SOFA at day three was given in only two studies, covering 267 patients. It was associated with higher in-ICU and in-hospital mortality. A Child–Pugh stage A was associated with lower in-ICU mortality. Results were heterogeneous for the Child–Pugh stage C. High MELD scores were associated with in-ICU mortality. In-hospital mortality was best predicted by a baseline MELD ≥ 13.

### Predictors of 6-month mortality in ICU survivors

**Fig. 4:**
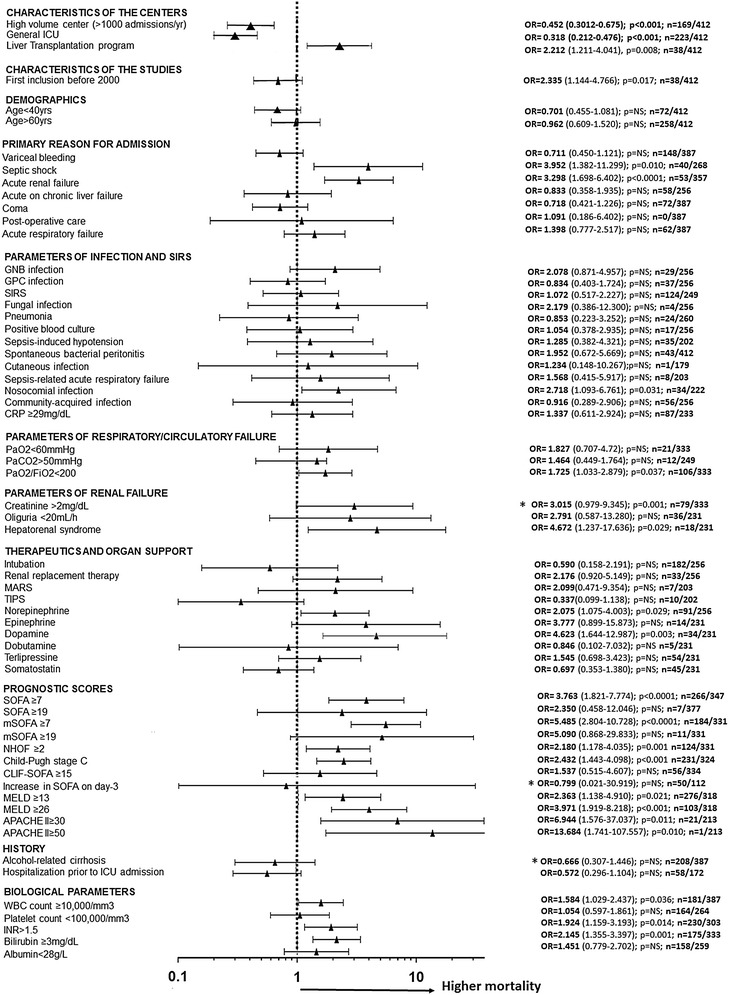
Predictors of 6-month mortality in ICU survivors. For each variable, combined weight-adjusted odds ratios (*filled triangle*) and their 95% confidence intervals (*horizontal line*) obtained from forest plots of pooled analyses are reported, together with the *p* value, the number of cirrhotic patients with variable present, and the total number of patients with available data. Odds ratios >1 correspond to variables associated with higher mortality. Odds ratios <1 correspond to variables associated with better survival. Odds ratios with a 95% CI containing 1 correspond to nonsignificant results. (*) indicates heterogeneous results. *APACHE* Acute Physiology and Chronic Health Evaluation, *CRP* C-reactive protein, *GNB* Gram-negative Bacilli, *GPB* Gram-positive Bacilli, *MARS* molecular adsorbents recirculation system, *MELD* model of end-stage liver disease, *NHOF* non-hematologic organ failure, *NS* not significant, *SIRS* systemic inflammatory response syndrome, *TIPS* transjugular intrahepatic portosystemic shunt, *SOFA* Sequential Organ Failure Assessment, *mSOFA* modified SOFA, *CLIF-SOFA* modified SOFA according to the Chronic Liver Failure Consortium of the European Association for the Study of the Liver

**Fig. 5:**
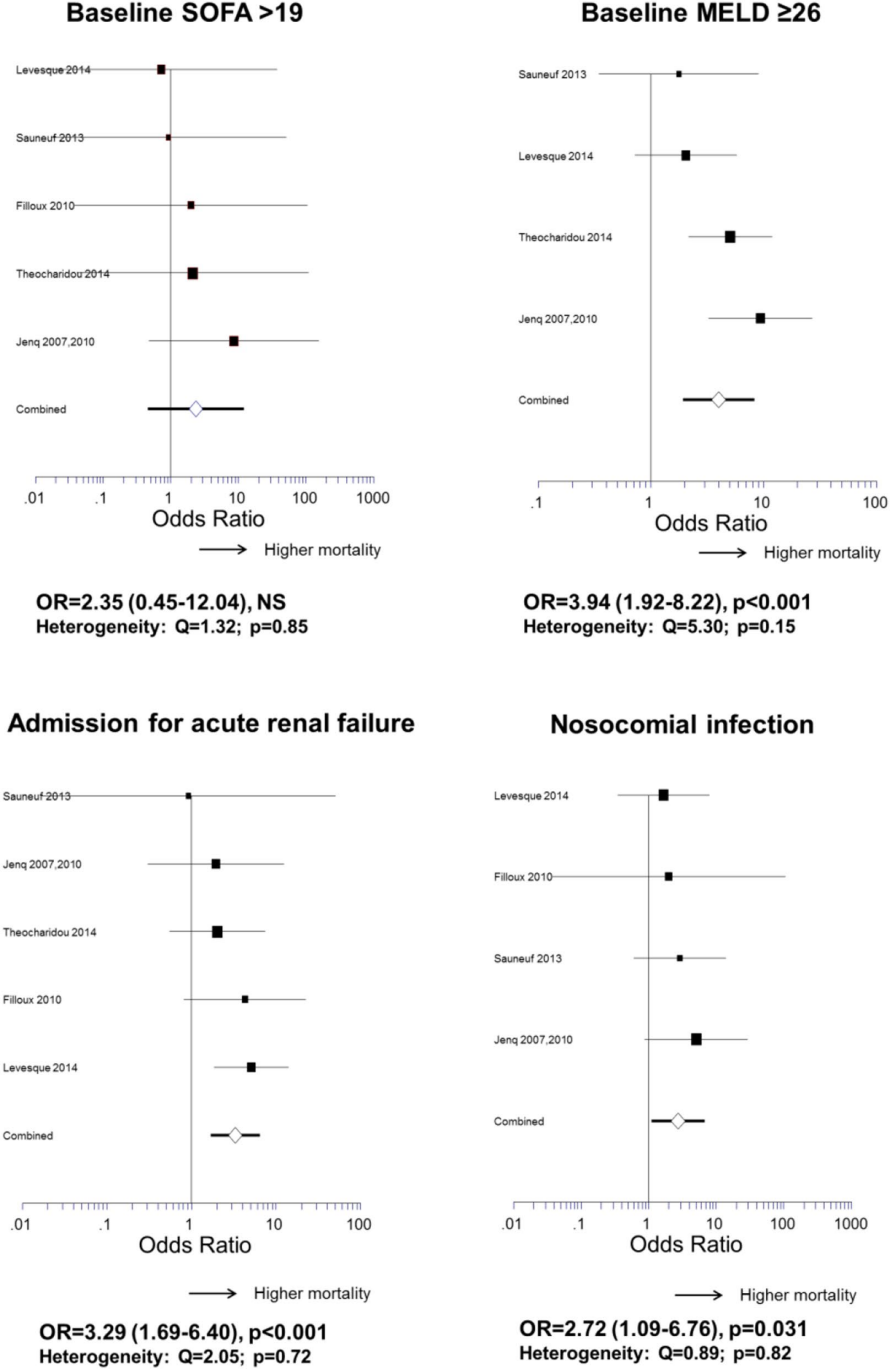
Some remarkable results regarding the impact of characteristics recorded on ICU admission on the 6-month mortality in ICU survivors (forest plots of odds ratios)

Variables with an impact on outcome of the 412 patients who survived the ICU phase are shown in Figs. 4 and 5 and Additional file 1: Table S4. General ICUs, high-volume centers, or centers with a routine TIPS program had lower mortality, whereas centers with a liver transplantation program had higher mortality. Age, sex, etiology of cirrhosis and duration of ICU stay had no significant influence. Parameters of liver function recorded on ICU admission such as bilirubin >3 mg/dL, INR > 2.3, MELD > 26, and Child–Pugh C stage had a strong influence. Renal impairment was also deleterious as assessed by hepatorenal syndrome prior to admission, renal failure as a reason for ICU admission, or need for renal replacement therapy. Other remarkable predictors were septic shock, nosocomial infection, and high leukocytes count at baseline. Among the ICU prognostic scores, high levels of SOFA did not impact on 6-month mortality, nor did an increase in SOFA at day three, conversely to high levels of CLIF-SOFA or APACHE-II.

## Discussion

Our meta-analysis investigated numerous characteristics of a large population of cirrhotic patients from different centers. It enabled us to provide original data regarding 6-month mortality in more than 400 patients who survived intensive care. It yields four major findings: (1) despite in-ICU mortality rates below 50% and decreasing in the more recent studies, medium-term prognosis remained poor and this finding was reproducible between studies. One possible explanation could be that a very small proportion of patients underwent liver transplantation; (2) short-term mortality was well predicted by ICU scores, which reflect the degree of organ dysfunction; (3) bad ICU score results at baseline did not predict 6-month mortality in ICU survivors. In those patients, the major determinants of outcome were liver and renal failure, underlining the value of the MELD; the CLIF-SOFA, that incorporates parameters of liver function, was the only score able to predict both in-ICU and mid-term survival; (4) some events considered alone, mostly related to SIRS and sepsis, were strong predictors of poor short-term prognosis, with performance similar to that of composite scores.

This analysis confirms that in-ICU survival was significantly better in patients admitted after 2004. This improvement in short-term prognosis over time suggests that substantial progress has been achieved in the management and/or selection of critically ill cirrhotic candidates for ICU. However, the dramatic decrease in medium-term survival illustrates the fragility of these patients and the limits of available efficient therapies beyond the initial control of organ failures allowing ICU discharge. Liver transplantation was rarely performed, even in centers with liver transplantation program available, and to date, this option appears to be inadequate to solve the problem. This finding is in keeping to what shown by a recent US study, in which only a small proportion of liver recipients (8.1%) comes from ICUs [41]. This is surprising, since liver transplantation of critically ill patients has been reported to provide acceptable results in some critically ill patients [42, 43]. Hence, the “sickest-first” policy applied in the USA and Europe to allocate liver grafts should give high priority to this population, providing the rigorous selection of patients in the context of organ shortage, by discarding those with disseminated hepatocellular carcinoma, severe comorbidities, uncontrolled sepsis, and presumed non-compliance, especially regarding alcohol consumption. All these points justify referring ICU survivors to a liver transplant center for careful evaluation.

In most studies, age was not reported to have an independent influence on mortality in this population, appearing only as a component of the APACHE score. In our study, one spectacular finding was the better prognosis of patients <40 years, who represent a small proportion of each cohort but can be adequately studied through meta-analysis. This finding is contrary to a recent study in which younger patients were supposed to have more severe acute on chronic liver failure (ACLF) [44]. Patients admitted to ICU for variceal bleeding had better short-term outcomes. This probably results from the more general use of pharmacological and endoscopic therapies, and TIPS, which efficiently control variceal bleeding. Pooled analyses of other reasons for admission suggested poor outcome, but were heterogeneous. Hence, indications other than variceal bleeding cannot be considered as deleterious *per se*. Similarly, results were heterogeneous regarding the impact of organ replacement therapy on ICU mortality. In particular, mechanical ventilation per se did not equal death, contrary to what Rabe et al. suggest [15]. The MARS system, perhaps because it was allocated to the sickest patients, was a strong surrogate for in-ICU mortality. This indicates that MARS is not powerful enough to cure multi-organ failure in cirrhotic patients, and should rather be considered as a bridge to liver transplantation.

Our study confirms the power of composite scores to predict in-ICU mortality. Among them, the SOFA was largely used and the recent CLIF-SOFA [45], although not used by the majority of authors at the time of their studies, provided strong predictions. The APACHE-II score was not widely used in cirrhotic patients, probably because of its complexity, and/or because of liver failure, which renders the interpretation confusing. These general scores were better than the liver-specific scores for the prediction of in-ICU mortality. It is acknowledged that short-term prognosis mostly depends on the number of organ failures and that scoring liver failure provides limited prognostic information in patients with multi-organ failure. Conversely, MELD and Child–Pugh, even measured in the context of organ failures, were able to predict 6-month mortality among ICU survivors. Interestingly, the APACHE-II and CLIF-SOFA, more influenced by liver failure than the SOFA, maintained their predictive value after ICU discharge. Moreover, we found that among the extrahepatic organ failures, renal failure was more powerful for the prediction of 6-month mortality in patients who survived ICU than for the prediction of in-ICU mortality. This is in keeping with results already reported by Fede et al. [14] and reinforces the value of the MELD score. The question of repeated evaluation of patients at day three, supported by some landmark papers [8, 46, 47], was also evaluated by our meta-analysis. Only a small proportion of patients (10%) had available SOFA at day three, thus decreasing the power of our pooled analyses. As a consequence, changes in SOFA between baseline and day 3 did not provide better predictions than baseline evaluations. This raises the question of the acceptability of this procedure, which supposes the interruption of organ support in the event of aggravation.

Another major result of our study is the impact of SIRS and sepsis in the outcome of cirrhotic patients admitted to ICU. The pejorative influence of SIRS and infection has been shown in non-ICU populations of cirrhotics [25, 48]. Our meta-analysis demonstrates that this is still true in the most severe cirrhotic patients regardless of the reason for admission in ICU. In particular, fungal infection, an event often associated with a delay in initiating the appropriate antimicrobial therapy, had a strong negative influence. Gram-negative infections were also significantly associated with mortality, highlighting the deleterious impact of endotoxinemia in the natural history of cirrhosis [25]. Pneumonia and its consequences were clearly a challenge for intensive care specialists, whereas spontaneous bacterial peritonitis, a surrogate of end-stage liver disease, had a deleterious influence in the longer term.

Our study acknowledges several limitations. First, the need of original data led to lack of exhaustiveness since a substantial proportion of authors was unable to share their data and answer our questionnaire. Second, because of missing data and/or recall biases, our meta-analyses of events that occurred during ICU stay were probably inaccurate. Third, among the 301 pooled analyses performed in the present work, 108 provided heterogeneous results and the source of heterogeneity was unknown. This is why we decided not to take heterogeneous results into account in our conclusions. Fourth, we did not study prognostic scores recently described, such as the quick SOFA [49] and the Besançon’s score [21], because they were not available at the time of study conception. The prognostic impact of ACLF was also incompletely studied because this condition was not defined at the time of some studies. Only a retrospective assessment of ACLF as a reason for admission could be studied, with no significant impact on mortality. Fifth, the design of this meta-analysis only allowed separate assessments of weight-adjusted pooled odds ratios for a single variable at different follow-up timepoints, but did not allow us to combine variables for analyses. However, correct multivariate analyses must include truly independent variables, and we assume that, in the context of multi-organ failures, clinical condition, biochemical parameters, and interventions are intricate. Sixth, because individual data were not available in the present study, it was not possible to provide new information regarding which patients could be considered futile for intensive care, and to confirm or not the conclusions from the CANONIC cohort recently published [44].

## Conclusions

Our analysis indicates that admission to ICU should be envisaged as soon as possible for critically ill cirrhotic patients, before too many extrahepatic failures compromise short-term survival. ICU survivors without heavy comorbidities can be identified early and may be systematically considered for transplantation. The number and intensity of organ failures on ICU admission does not mask the power of MELD score measured concomitantly for predicting 6-month mortality and thus immediately discriminates the best candidates for liver transplantation. The CLIF-SOFA is relevant for discriminating both in-ICU and mid-term mortality in critically ill cirrhotic patients.

## Study highlights

### What is already known on this subject?

-  The outcome of cirrhotic patients admitted to ICUs is poor but varies between centers.
-  Numerous prognostic scores have been tested in this situation, only focused on short-term mortality.

### What are the new findings?

- We report the predictors of mid-term survival in patients who survive the ICU phase.
-  We observe a good predictive value of MELD score measured at baseline (ICU admission), even in the context of multi-organ failures, to predict mid-term mortality.

### How might it impact on clinical practice in the foreseeable future?

- Early discrimination of patients who will survive the ICU phase but with remaining risk of mortality after discharge will accelerate the access to liver transplantation of the good candidates, today not systematically considered.

## Additional file

**Additional file 1.** **Table S1.** Patients characteristics on admission. **Table S2.** Predictors of in-ICU mortality. **Table S3.** Predictors of in-hospital mortality. **Table S4.** Predictors of 6-month mortality in ICU survivors. **Figure S1.** Flow diagram. Literature search and selection process.

## Abbreviations

95% CI: 95% confidence interval
ACLF: acute-on-chronic liver failure
APACHE: Acute Physiology and Chronic Health Evaluation
CRP: C-reactive protein
ICU: intensive care unit
INR: international normalized ratio
MARS: molecular adsorbents recirculation system
MELD: model for end-stage liver disease
NA: not available
NHOF: non-hematologic organ failure
NPV: negative predictive value
NS: not significant
OR: odds ratio
PPV: positive predictive value
PRISMA: preferred reporting items for systematic reviews and meta-analyses
SOFA: Sequential Organ Failure Assessment
SIRS: systemic inflammatory response syndrome
TIPS: transjugular portosystemic shunt
