# Supplementary material for: Prognosis of cirrhotic patients admitted to intensive care unit: a meta-analysis
Source: Ann Intensive Care. 2017 Mar 21;7:33. doi: 10.1186/s13613-017-0249-6 (PMC5359266; doi:10.1186/s13613-017-0249-6)
Supplement: Supplementary file 1 — Additional file 1. Table S1. Patients characteristics on admission. Table S2. Predictors of in-ICU mortality. Table S3. Predictors of in-hospital mortality. Table S4. Predictors of 6-month mortality in ICU survivors. Figure S1. Flow diagram. Literature search and selection process. [file 13613_2017_249_MOESM1_ESM.doc]

Table S1: Patients characteristics on admission

|  | **Jenq^[30,31]^ (n=188)** | **Fichet^[10]^ (n=71)** | **Sauneuf^[5]^**  **(n=89)** | **Das^[8]^**  **(n=138)** | **Galbois^[6]^**  **(n=56)** | **Levesque^[2,3]^ (n=451)** | **Theocharidou^[7]^**  **(n=265)** | **Filloux^[29]^**  **(n=83)** | **Cavallazzi^[28]^**  **(n=441)** | **Karvellas^[32]^**  **(n=178)** | **Shawcross^[33]^**  **(n=563)** | **% TOTAL** |
| --- | --- | --- | --- | --- | --- | --- | --- | --- | --- | --- | --- | --- |
| **SEX**  Male (n(%)) | 138(73.4) | 53(74.6) | 62(69.6) | 94(68.1) | 31(55.3) | 335(74.2) | 174(65.6) | 64(77.1) | 281(63.7) | 109(61.2) | 348(61.8) | 1689(66.9) |
| **AGE (n(%))**  <40 years  <50 years  <60 years  >70 years | 18(9.6)  62(33.0)  105(55.9)  36(19.1) | 4(5.6)  14(19.7)  38(53.5)  12(16.9) | 3(3.4)  22(24.7)  55(61.8)  13(14.6) | 9(6.5)  37(26.8)  92(66.7)  18(13.0) | 6(10.7)  19(33.9)  36(64.3)  13(23.2) | 37(8.2)  141(31.2)  291(64.5)  32(7.1) | 38(14.3)  102(38.5)  188(70.9)  31(11.7) | 3(3.6)  17(20.5)  49(59.0)  11(13.2) | 18(4.1)  83(18.8)  259(58.7)  79(17.9) | 39(21.9)  91(51.1)  144(80.9)  1(0.5) | 126(22.4)  273(48.5)  396(70.3)  62(11.0) | 382(15.1)  861(34.1)  1653(65.5)  308(12.2) |
| **STAY DURATION (n(%))**  <3 days  >14 days | 45(23.9)  30(16.0) | 24(33.8)  10(14.1) | 23(25.8)  34(38.2) | 41(29.7)  16(11.6) | 14(25.0)  4(7.1) | 126(27.9)  94(20.8) | 119(44.9)  34(12.8) | 14(16.8)  22(26.5) | -  - | 32(18)  84(47.2) | 95(16.9)  163(28.9) | 533(26.1)  491(23.6) |
| **ETIOLOGY OF CIRRHOSIS (n(%))**  Alcohol  Virus  NASH  Other | 76(40.4)  87(46.3)  12(6.4)  4(2.1) | 53(74.6)  5(7.0)  -  13(18.3) | 72(80.9)  8(9.0)  -  9(10.1) | 104(75.4)  20(14.5)  -  11(8.0) | 42(75.0)  6(10.7)  -  8(14.3) | 331(73.4)  79(17.5)  -  - | 180(67.9)  13(4.9)  -  72(27.2) | 73(87.9)  4(4.8)  1(1.2)  5(6.0) | 203(46.0)  141(32.0)  23(5.2)  74(16.8) | 77(43.3)  23(12.9)  26(14.6)  - | 263(46.7)  98(17.4)  16(2.8)  186(33.0) | 1474(58.4)  529(20.9)  88(5.8)  388(20.5) |
| **PATIENTS’ ORIGIN**  Hospitalized (n(%)) | 56(29.8) | - | 30(33.7) | 78(56.5) | 29(51.8) | - | - | 38(45.7) | - | - | - | 231(41.6) |
| **PRIMARY REASON**  **FOR ADMISSION**  Variceal bleeding  Septic shock  Acute renal failure  Acute Lung Injury  Coma  Post-operative management  ACLF | 62(33.0)  9(4.8)  6(3.2)  18(9.6)  39(20.7)  0(0.0)  31(16.5) | 4(5.6)  2(2.8)  -  1(1.4)  54(76.1)  1(1.4)  - | 0  89(100)  0  0  0  0(0.0)  - | 24(17.4)  40(29.0)  5(3.6)  37(26.8)  32(23.2)  0(0.0)  - | 11(19.6)  13(23.2)  7(12.5)  15(26.8)  10(17.9)  0(0.0)  - | 184(40.8)  61(13.5)  220(48.8)  119(26.3)  108(23.9)  -  330(73.2) | 149(56.2)  -  17(6.4)  20(7.5)  21(7.9)  0(0.0)  - | 10(12.0)  10(12.0)  13(15.6)  23(27.7)  19(22.9)  0(0.0)  8(9.6) | 113(25.6)  -  25(5.7)  87(19.7)  -  -  - | -  -  -  -  19(10.7)  -  - | 206(36.6)  11(20.4)  41(7.3)  22(3.9)  103(18.3)  7(1.2)  - | -  -  -  -  -  -  - |
| **RENAL FAILURE** |  |  |  |  |  |  |  |  |  |  |  |  |
| Hepato-renal syndrome | 27(14.4) | - | - | - | 2(3.6) | 19(5.0) | - | 6(7.2) | - | 86(48.3) | 239(42.5) | 379(21.3) |
| Oliguria <20 mL/H | 78(41,5) | - | - | 41(29,7) | 16(28,6) | 107(23.7) | - | 9(10,8) | - | - | 187(33,2) | 438(29.6) |
| Creatinine > 132µmol/L | 135(71.8) | - | 47(52.8) | 85(61.6) | 21(37.5) | 177(39.2) | 84(31.7) | 35(42.1) | 238(54.0) | 94(52.8) | 280(49.7) | 1196(48.8) |
| **NEUROLOGICAL FAILURE** |  |  |  |  |  |  |  |  |  |  |  |  |
| Encephalopathy (all stages) | 167(88.8) | - | - | 87(63.0) | 28(50.0) | 266(58.9) | - | 83(100.0) | - | - | 376(66.8) | 1007(68.1) |
| Coma (GCS<8) | 64(34.0) | - | - | 57(41.3) | 12(21.4) | 67(14.8) | 167(63.0) | 21(25.3) | - | 19(10.7) | 69(12.3) | 476(24.7) |
| **RESPIRATORY FAILURE** |  |  |  |  |  |  |  |  |  |  |  |  |
| PaO2/FiO2<200 | 51(27.1) | - | - | 21(15.2) | 5(8.9) | 81(17.9) | 125(47.2) | - | - | 83(46.6) | 261(46.4) | 627(34.1) |
| PaO2<60mmHg | 15(8.0) | - | - | - | - | 2(0.4) | 39(14.7) |  |  | 2(1.1) | 66(11.7) | 124 (7.5) |
| pH<7.3 | 53(28.2) | - | - | - | - | - | 77(29.1) | - | - | 47(26.4) | 137(24.3) | 314 (26.3) |
| **INFLAMMATION/ INFECTION** |  |  |  |  |  |  |  |  |  |  |  |  |
| ≥ 2 criteria for SIRS | 131(69.7) | - | 89(100.0) | - | - | - | 105(39.6) | - | - | 89(50.0) | - | 414(57.5) |
| WBC >10000/mm^3^ | 107(56.9) | - | 52(58.4) | - | - | 251(55.6) | 109(41.1) | 52(62.6) | - | 86(48.3) | 239(42.5) | 856(62.6) |
| WBC <4000/mm^3^ | 12(6.4) | - | 10(11.2) | - | - | 22(4.8) | 18(6.8) | 12(14.4) | - | - | 66(11.7) | 140(11.3) |
| CRP >10mg | 144(76.6) | - | 82(92.1) | - | - | - | 172(64.9) | - | - | - | - | 398(73.4) |
| CRP >29mg | 88(46.8) | - | 75(84.3) | - | - | - | 95(35.8) | - | - | - | - | 258(47.6) |
| Infection  Nosocomial  Community-acquired | 33(17.6)  63(33.5) | -  - | 27(30.3)  62(69.7) | 5(3.6)  - | -  - | 17(3.7)  88(19.5) | -  - | 0(0.0)  6(7.0) | -  - | -  - | -  - | 82(8.6)  211(26.0) |
| Sepsis-induced hypotension | 28(14.9) | - | 89(100.0) | 41(29.7) | - | 62(13.7) | - | - | - | - | - | 220(25.4) |
| Pneumonia | 18(9.6) | - | 37(41.6) | 23(16.7) | 13(23.2) | 59(13.1) | - | 0 | - | - | - | 137(13.6) |
| Pneumonia-induced ALI | 4(2.1) | - | 18(20.2) | 24(17.4) | 3(5.4) | 49(10.8) | - | - | - | - | - | 98(10.6) |
| Spontaneous bacterial peritonitis | 43(22.9) | - | 22(24.7) | 10(7.2) | 4(7.1) | 25(5.5) | 30(11.3) | 0 | - | - | - | 134(10.5) |
| Urinary tract infection | 20(10.6) | - | 10(11.2) | 3(2.2) | - | 8(1.7) | - | 0(0.0) | - | - | - | 41(2.3) |
| Other infection | 12(6.4) | - | 20(22.5) | 12(8.7) | 4(7.1) | 0(0.0) | - | - | - | - | - | 48(5.2) |
| ≥ 1 positive blood culture | 19(10,1) | - | 34(38.2) | - | - | 24(5.3) | - | 0 | - | 65(36.5) | - | 142(14.3) |
| GNB | 44(23.4) | - | 35(39.3) | - | - | 33(7.3) | - | 6(7.2) | - | - | - | 118(14.5) |
| GBP | 10(5.3) | - | - | - | - | 10(2.2) | - | 0(0.0) | - | - | - | 20(2.7) |
| GPC | 35(18.6) | - | 35(39.3) | - | - | 12(2.6) | - | 8(9.6) | - | - | - | 90(11.1) |
| Fungal | 7(3.7) | - | 4(4.5) | - | - | 21(4.6) | - | 1(1.2) | - | - | - | 33(4.0) |
| Lactates upper normal value | 19(10.1) | - | 68(76.4) | - | - | 124 (16.7) | 147(55.5) | - | - | 83(46.6) | 295(92.4) | 736(42.4) |
| **OTHER PARAMETERS** |  |  |  |  |  |  |  |  |  |  |  |  |
| Ascites | 136(72.3) | - | - | 71(51.4) | 24(42.9) | 162(35.9) | - | - | - | - | 510(90.6) | 733(52.5) |
| Mean Arterial Pressure <65mmHg | 31(16.5) | - | - | 43(31.2) | - | 73(16.2) | 30(11.3) | - | - | 83(46.6) | 260(46.2) | 520(34.2) |
| Albumin <28g/L | 146(77.7) | 37(52.1) | - | 86(62.3) | 22(39.3.) | 48(10.6) | 181(68.3) | - | - | - | 393(69.8) | 913(52.7) |
| Platelets<100000/µL | 128(68.1) | - | - | 34(24.6) | 25(44.6) | 94(20.8) | 162(61.1) | - | 244(55.3) | - | 378(67.1) | 1065(50.6) |
| Bilirubin ≥51µmol/L | 135(71.8) | - | 47(52.88) | 83(60.1) | 22(39.3) | 289(64.1) | 162(61.1) | - | 245(55.6) | 125(70.2) | 387(68.7) | 1495(63.1) |
| Natremia <125mmol/L | 18(9.6) | 9(12.7) | 7(7.9) | 13(9.4) | 7(12.5) | 38(8.4) | 23(8.7) | 7(8.4) | 17(3.9) | - | 75(13.3) | 214(9.1) |
| INR >2,3 | 91(48.4) | 34(47.9) | 43(48.3) | 39(28.3) | 15(26.8) | 221(49.0) | 74(27.9) | 34(41.0) | 110(24.9) | 25(14.0) | 88(15.6) | 774(30.6) |
| **SCORES (n(%))** |  |  |  |  |  |  |  |  |  |  |  |  |
| CHILD  A  B  C | 4(2.1)  36(19.1)  144(76.6) | 8(11.3)  9(12.7)  54(76.1) | 8(9.0)  32(36.0)  49(55.0) | 10(7.2)  38(27.5)  90(65.2) | 13(23.2)  17(30.4)  26(46.4) | 26(5.7)  96(21.3)  329(72.9) | 7(2.6)  77(29.1)  166(62.6) | -  -  - | -  -  - | 5(2.8)  43(24.2)  130(73.0) | 4(0.7)  63(11.2)  496(88.1) | 85(4.2)  411(20.5)  1484(74.2) |
| MELD  <13  <18  >25 | 8(4.3)  44(23.4)  86(45.7) | -  -  - | 10(11.2)  21(23.6)  41(46.1) | 18(13.0)  52(37.7)  49(35.5) | 17(30.4)  31(55.4)  15(26.8) | 54(11.9)  120(26.6)  232(51.4) | 22(8.3)  70(26.4)  79(29.8) | -  -  - | 59(13.4)  140(31.7)  185(42.0) | 31(17.4)  49(27.5)  97(54.5) | 128(22,7  189(33,6)  271(48,2) | 347(14.6)  716(30.2)  1055(44.5) |
| SOFA  <7  <13  >19 | 40(21.3)  154(81.9)  17(9.0) | -  -  - | 11(12.4)  39(43.8)  9(10.1) | 34(24.6)  80(58.0)  16(11.6) | 31(55.4)  43(76.8)  3(5.4) | 136(30.1)  315(69.8)  29(6.4) | 52(19.6)  179(67.5)  1(0.4) | 7(8.4)  60(72.3)  6(7.2) | -  -  - | -  -  - | 81(14.4)  377(67.0)  29 (5.2) | 392(21.4)  1247(68.0)  110(6.0) |
| mSOFA  <6  >13  ≥19 | 70(37.2)  54(28,7)  0 (0.0) | -  -  - | 21(23.6)  42(47,1)  2(2.2) | 50(36.2)  67(48,5)  0(0.0) | 36(64.3)  9(16.1)  0(0.0) | 195(43.2)  95(21.0)  32(7.1) | 131(49.4)  10(3.8)  0(0.0) | -  -  - | -  -  - | -  -  - | -  -  - | 503(42.4)  277(23.3)  34(2.8) |
| CLIF-SOFA  <14  >22 | 154(81.9)  0(0.0) | -  - | 38(42.7)  4(4.5) | -  - | 45(80.4)  1(1.8) | 316(70.0)  25(5.5) | 216(81.5)  1(0.4) | -  - | -  - | -  - | -  - | 769(73.3)  31(2.8) |
| APACHE II  <20  ≥ 40 | 50(20,6)  16(8,5) | -  - | -  - | -  - | -  - | -  - | 192(72,5)  1(0,4) | -  - | -  - | 70(39.3)  6(3.4) | 201(38,1)  13(2,4) | 513(42.9)  36(3.0) |
| NHOF  >2  >3  >4 | 111(59.4)  65(34.5)  31(16.5) | -  -  - | 80(89.9)  25(28.1)  16(18.0) | 76(50.1)  56(40.6)  81(58.7) | 18(32.1)  12(21.4)  8(14.3) | 178(39.4)  98(21.7)  52(11.5) | 100(37.7)  22(8.3)  2(0.7) | -  -  - | -  -  - | -  -  - | -  -  - | 563(47.4)  329(23.4)  227(16.0) |
| **THERAPEUTICS USED DURING ICU STAY**  INTUBATION  RRT  MARS  TIPS  NOREPINEPHRINE  EPINEPHRINE  DOBUTAMINE  DOPAMINE  TERLIPRESSINE  OCTREOTIDE | 139(72.9)  34(18.1)  1(0.5)  0(0)  51(27.1)  59 (31.4)  2 (1.1)  109(57.9)  109(57.9)  15(7.9) | -  -  -  -  -  -  -  -  -  - | 85(95.5)  56(62.9)  0(0)  0(0)  89(100)  -  -  -  -  - | 85(61.6)  26(18.8)  2(1.4)  -  71(51.4)  -  -  -  -  - | 29(51.8)  7(12.5)  0(0)  -  5(8.9)  -  -  -  -  - | 246(54.5)  50(11.1)  45(9.9)  44(9.7)  207(45.9)  7(1.5)  15(3.3)  2(0.4)  21(4.6)  184(40.7) | -  -  -  -  -  -  -  -  -  - | 50(60.2)  16(19.2)  -  -  38(45.8)  0(0)  12(14.5)  0(0)  4(4.8)  11(13.2) | 283(64.2)  93(21.1)  -  -  -  -  -  -  -  - | 134(75.3)  96(53.9)  -  -  47(26.4)  -  -  -  -  - | 41(974.4)  274(48.6)  -  -  271(48.1)  -  -  -  -  - | 1468(67.1)  652(29.8)  46(5.8)  44(6.0)  779(44.6)  66(9.1)  29(4.0)  111(15.3)  134(18.5)  210 (29.1) |

Table S2: Predictors of in-ICU mortality

| **Variables** | **N studies** | **N patients *** | **Weight-adjusted OR (95%CI)** | **P** | **Heterogeneity**  **Q ; p** | **PPV (95%CI)** | **NPV (95%CI)** |
| --- | --- | --- | --- | --- | --- | --- | --- |
|  |  |  |  |  |  |  |  |
| **DEMOGRAPHIS** |  |  |  |  |  |  |  |
| Female | 10 | 605/1904 | 1.07 (0.85-1.33) | NS | 5.73; 0.67 | - | - |
| Age>40 | 10 | 1660/1904 | 1.41 (1.06-1.87) | 0.019 | 4.95; 0.83 | 0.46 (0.43-0.48) | 0.63 (0.58-0.69) |
| Age>50 | 10 | 1217/1904 | 1.32 (1.09-1.62) | 0.005 | 7.54; 0.48 | 0.47 (0.44-0.50) | 0.59 (0.56-0.63) |
| Age>60 | 10 | 611/1904 | 1.12 (0.92-1.37) | NS | 3.01; 0.93 | - | - |
| Age>70 | 10 | 182/1904 | 1.27 (0.93-1.73) | NS | 1.43; 0.99 | - | - |
| **ETIOLOGY OF CIRRHOSIS** |  |  |  |  |  |  |  |
| Alcohol | 10 | 1197/1904 | 0.98 (0.73-1.32) | NS | 13.59; 0.09 | - | - |
| Virus | 10 | 320/1898 | 1.25 (0.85-1.84) | NS | 13.18; 0.11 | - | - |
| Metabolic | 6 | 29/1050 | 0.74 (0.37-1.48) | NS | 0.45; 0.99 | - | - |
| **HISTORY OF CIRRHOSIS** |  |  |  |  |  |  |  |
| Past History Of Ascites | 3 | 191/382 | 1.21 (0.63-2.33) | NS | 3.52; 0.17 | - | - |
| Past History Of Curable HCC | 2 | 18/277 | 0.19 (0.01-7.32) | NS | 5.47; 0.02 | - | - |
| Past History Of Incurable HCC | 4 | 37/471 | 1.11 (0.57-2.15) | NS | 0.36; 0.95 | - | - |
| Past History Of Variceal Bleeding | 3 | 103/382 | 0.90 (0.45-1.80) | NS | 4.109-0.128 | - | - |
| **MAIN REASON FOR ADMISSION** |  |  |  |  |  |  |  |
| Variceal Bleeding | 10 | 650/1904 | 0.46 (0.36-0.59) | <0.001 | 9.44; 0.31 | 0.30 (0.26-0.33) | 0.50 (0.48-0.53) |
| Post Operative Management | 10 | 8/1879 | 1.02 (0.36-2.84) | NS | 3.66; 0.88 | - | - |
| Acute Respiratory Failure | 10 | 255/1904 | 1.73 (0.79-3.81) | NS | 41.44; <0.01 | - | - |
| Septic Shock | 9 | 339/1639 | 2.87 (1.58-5.21) | <0.001 | 18.47; 0.01 | 0.69 (0.64-0.74) | 0.62 (0.60-0.65) |
| Acute Renal Failure | 10 | 309/1904 | 1.49 (0.75-3.75) | NS | 43.02; <0.01 | - | - |
| Acute-On-Chronic Liver Failure | 6 | 369/882 | 3.61 (0.8016.28) | NS | 19.42; <0.01 |  |  |
| Coma | 10 | 383/1892 | 1.08 (0.53-1.96) | NS | 37.37; <0.01 |  |  |
| **HOSPITALIZATION PRIOR TO ICU ADMISSION** | 5 | 231/554 | 1.03 (0.53-1.99) | NS | 12.69; 0.01 | - | - |
| **CHARACTERISTICS ON ADMISSION** |  |  |  |  |  |  |  |
| Ascites | 5 | 903/1322 | 1.71 (0.67-4.34) | NS | 39.8; <0.01 | - | - |
| Hepatorenal Syndrome | 5 | 56/779 | 1.26 (0.72-2.21) | NS | 2.60; 0.45 | - | - |
| **Parameters Of SIRS/Infection** |  |  |  |  |  |  |  |
| SIRS According To Heart Rate | 5 | 585/1155 | 1.19 (0.63-2.23) | NS | 20.55; <0.01 | - | - |
| SIRS According To Respiratory Rate | 3 | 249/536 | 18.13 (1.06-12.42) | <0.001 | 14.89; <0.01 | 0.52 (0.46-0.58) | 0.70 (0.65-0.75) |
| SIRS According To Body Temperature | 4 | 423/1099 | 2.41 (1.65-3.51) | <0.001 | 4.27; 0.23 | 0.55 (0.51-0.60) | 0.65 (0.61-0.68) |
| ≥2 Criteria For SIRS | 3 | 325/542 | 2.44 (1.64-3.65) | <0.001 | <0.01; 0.99 | 0.57 (0.52-0.63) | 0.71 (0.65-0.77) |
| Infection | 6 | 301/949 | 2.59 (1.04-6.47) | <0.001 | 12.67; 0.01 | 0.65 (0.60-0.71) | 0.66 (0.62-0.69) |
| Nosocomial Infection | 6 | 82/879 | 0.96 (0.22-4.19) | NS | 24.48; <0.01 | - | - |
| Community-Acquired Infection | 5 | 228/820 | 2.34 0.99-5.80) | NS | 13.53; 0.003 | - | 6 |
| GNB Infection | 5 | 118/811 | 2.24 (1.46-3.43) | <0.001 | 2.72; 0.44 | 0.68 (0.59-0.76) | 0.59 (0.55-0.63) |
| GPB Infection | 4 | 20/747 | 2.68 (0.46-15.69) | NS | 5.12; 0.16 | - | - |
| GPC Infection | 5 | 90/811 | 0.81 (0.45-1.45) | NS | 3.60; 0.31 | - | - |
| Fungal Infection | 5 | 33/811 | 4.38 (1.11-17.24) | <0.001 | 5.73; 0.12 | 0.85 (0.68-0.95) | 0.57 (0.53-0.60) |
| Pneumonia | 7 | 150/925 | 2.18 (1.47-3.22) | <0.001 | 5.14; 0.39 | 0.69 (0.61-0.77) | 0.46 (0.43-0.50) |
| Urinary Tract Infection | 6 | 41/949 | 1.52 (0.81-2.88) | NS | 2.90; 0.57 | - | - |
| Spontaneous Bacterial Peritonitis | 8 | 134/1290 | 1.59 (0.72-3.52) | NS | 20.18; <0.01 | - | - |
| Infection Of Unusual Site | 7 | 53/1047 | 2.53 (1.27-5.04) | 0.003 | 7.11; 0.31 | 0.09 (0.06-0.12) | 0.97 (0.96-0.99) |
| Cutaneous Infection | 4 | 6/818 | 1.48 (0.39-5.64) | NS | 0.49; 0.92 | - | - |
| Positive Blood Culture | 5 | 77/811 | 2.03 (1.00-4.12) | 0.008 | 4.62; 0.20 | 0.70 (0.59-0.80) | 0.58 (0.54-0.62) |
| Sepsis-Induced Hypotension | 5 | 220/866 | 6.63 (4.26-10.34) | <0.001 | 1.80; 0.61 | 0.75 (0.69-0.81) | 0.65 (0.61-0.69) |
| Sepsis-induced refractory oliguria* | 5 | 187/833 | 10.61 (4.07-27.63) | <0.001 | 9.39; 0.20 | 0.76 (0.70-0.82) | 0.68 (0.64-0.72) |
| Pneumonia-Induced Acute Respiratory Failure | 6 | 98/922 | 6.57 (3.89-11.09) | <0.001 | 2.22; 0.69 | 0.83 (0.74-0.90) | 0.60 (0.56-0.63) |
| **Parameters Of Renal failure** |  |  |  |  |  |  |  |
| Creatinine ≥ 1.5mg/dL | 9 | 862/1833 | 3.81 (2.57-5.64) | <0.001 | 20.80; <0.01 | 0.61 (0.58-0.65) | 0.73 (0.70-0.76) |
| Use Of Nephrotoxic Drug | 2 | 54/277 | 1.59 (0.63-3.97) | NS | 1.28; 0.26 | - | - |
| Oliguria ≤20 mL/h | 7 | 438/1479 | 5.68 (3.61-8.93) | <0.001 | 12.46; 0.03 | 0.73 (0.68-0.77) | 0.70 (0.67-0.72) |
| Creatinine>2.0 mg/dL | 8 | 344/1626 | 4.21 (2.26-7.85) | <0.001 | 32.65; <0.01 | 0.67 (0.62-0.71) | 0.66 (0.64-0.69) |
| **Parameters Of Respiratory/Circulatory Failure** |  |  |  |  |  |  |  |
| Pao2<60mmHg | 6 | 122/1556 | 2.08 (1.41-3.06) | <0.001 | 3.22; 0.52 | 0.59 (0.50-0.68) | 0.58 (0.55-0.60) |
| Paco2>50mmHg | 3 | 36/542 | 2.57 (1.31-5.05) | 0.006 | 1.29; 0.52 | 0.61 (0.45-0.77) | 0.55 (0.51-0.59) |
| Ph<7.3 | 4 | 267/1177 | 1.71 (0.92-3.18) | <0.001 | 9.74; 0.02 | 0.58 (0.53-0.64) | 0.53 (0.50-0.56) |
| Pao2/Fio2<200 | 8 | 547/1750 | 2.46 (1.47-4.10) | <0.001 | 21.20; <0.01 | 0.57 (0.53-0.62) | 0.63 (0.60-0.66) |
| Pao2/Fio2<100 | 8 | 150/1749 | 2.37 (1.63-3.45) | <0.001 | 6.19; 0.40 | 0.62 (0.53-0.69) | 0.58 (0.56-0.61) |
| Mean Arterial Pressure<65mmHg | 7 | 437/1694 | 2.27(1.27-4.04) | <0.001 | 19.89; <0.01 | 0.58 (0.53-0.63) | 0.61 (0.59-0.64) |
| **Parameters Of Neurological failure** |  |  |  |  |  |  |  |
| Hepatic Encephalopathy Stage 1 | 7 | 432/1479 | 1.12 (0.63-2.01) | NS | 21.17; <0.01 | - | - |
| Hepatic Encephalopathy Stage 2 | 7 | 250/1479 | 1.50(0.95-2.37) | NS | 11.18; 0.04 | - | - |
| Hepatic Encephalopathy Stage 3 | 7 | 325/1479 | 3.02 (1.88-4.84) | <0.001 | 12.82; 0.02 | 0.66 (0.59-0.72) | 0.64 (0.61-0.66) |
| Glasgow Coma Scale ≤7 | 7 | 491/1815 | 2.69 (1.62-4.48) | <0.001 | 26.81; <0.01 | 0.56 (0.51-0.60) | 0.64 (0.61-0.66) |
| Glasgow Coma Scale ≤12 | 7 | 872/1662 | 2.86 (1.59-5.16) | <0.001 | 38.43; <0.01 | 0.56 (0.53-0.60) | 0.74 (0.70-0.77) |
| **Biochemical parameters** |  |  |  |  |  |  |  |
| Bilirubin>2mg/dL | 8 | 1149/1751 | 2.52 (1.63-3.91) | <0.001 | 17.34; 0.01 | 0.52 (0.49-0.55) | 0.68 (0.64-0.71) |
| Bilirubin>3mg/dL | 8 | 1125/1751 | 3.34 (2.68-4.17) | <0.001 | 2.35; 0.88 | 0.53 (0.50-0.56) | 0.74 (0.70-0.77) |
| Platelet Count <100,000/mm3 | 6 | 807/1639 | 1.78 (0.96-3.28) | NS | 27.39; <0.01 |  |  |
| INR >1.5 | 8 | 889/1750 | 3.82 (2.52-5.80) | <0.001 | 17.28; <0.01 | 0.58 (0.55-0.61) | 0.72 (0.69-0.75) |
| INR >2.3 | 10 | 639/1858 | 3.59 (2.62-4.93) | <0.001 | 14.07; 0.08 | 0.61 (0.57-0.65) | 0.66 (0.63-0.69) |
| White Blood Cell Count>10,000/mm3 | 7 | 810/1639 | 1.50 (1.22-1.83) | <0.001 | 4.69; 0.45 | 0.48 (0.45-0.52) | 0.62 (0.58-0.65) |
| White Blood Cell Count <4,000/mm3 | 7 | 140/1639 | 1.38 (0.63-2.98) | NS | 15.07; 0.01 |  |  |
| CRP>29mg/L | 3 | 258/542 | 1.82 (1.26-2.62) | 0.001 | 1.79; 0.41 | 0.57 (0.51-0.63) | 0.64 (0.58-0.70) |
| Albumin<28g/L | 7 | 913/1715 | 2.16 (1.22-3.82) | <0.001 | 28.80; <0.01 | 0.47 (0.44-0.51) | 0.65 (0.62-0.68) |
| Natremia<125 mmol/L | 10 | 197/1904 | 1.40 (1.04-1.87) | 0.025 | 4.79; 0.78 | 0.50 (0.43-0.57) | 0.58 (0.56-0.60) |
| Lactate Upper Normal Value | 6 | 653/1556 | 3.23 (2.17-4.79) | <0.001 | 9.08; 0.06 | 0.59 (0.55-0.63) | 0.67 (0.64-0.70) |
| **Therapeutics** |  |  |  |  |  |  |  |
| Intubation | 8 | 1052/1568 | 12.07 (5.12-28.41) | <0.001 | 32.75; <0.01 | 0.61 (0.58-0.64) | 0.90 (0.87-0.92) |
| Renal Replacement Therapy | 8 | 463/1588 | 4.41 (2.03-9.58) | <0.001 | 36.05; <0.01 | 0.73 (0.69-0.77) | 0.68 (0.65-0.71) |
| MARS | 7 | 55/1485 | 2.07 (1.22-3.53) | 0.007 | 1.16; 0.94 | 0.58 (0.44-0.71) | 0.55 (0.53-0.58) |
| TIPS | 4 | 44/728 | 0.64 (0.33-1.23) | NS | 0.10; 0.95 | - | - |
| Norepinephrine | 8 | 748/1568 | 16.78 (8.29-33.95) | <0.001 | 26.52; <0.01 | 0.76 (0.73-0.79) | 0.84 (0.82-0.87) |
| Epinephrine | 4 | 66/722 | 5.03 (2.68-9.42) | <0.001 | 0.54; 0.76 | 0.77 (0.65-0.87) | 0.62 (0.58-0.66) |
| Dobutamine | 4 | 29/722 | 8.92 (3.32-23.96) | <0.001 | 0.80; 0.67 | 0.86 (0.68-0.96) | 0.60 (0.57-0.64) |
| Glypressine | 4 | 111/722 | 1.45 (0.91-2.31) | NS | 0.29; 0.86 | - | -- |
| Dopamine | 4 | 134/722 | 5.57 (3.02-10.27) | <0.001 | 1.64; 0.43 | 0.68 (0.59-0.77) | 0.64 (0.60-0.67) |
| Somatostatin | 4 | 210/722 | 0.43 (0.30-0.63) | <0.001 | 1.39; 0.49 | 0.27 (0.21-0.34) | 0.53 (0.48-0.57) |
| **Scores** |  |  |  |  |  |  |  |
| SOFA>7 | 9 | 1438/1813 | 7.45 (3.53-15.71) | <0.001 | 23.64; <0.01 | 0.52 (0.49-0.54) | 0.90 (0.87-0.93) |
| SOFA >13 | 9 | 598/1813 | 15.13 (4.73-48.38) | <0.001 | 38.84; <0.01 | 0.76 (0.72-0.79) | 0.74 (0.71-0.76) |
| SOFA >19 | 9 | 113/1813 | 8.54 (2.09-34.91) | <0.001 | 11.16; 0.05 | 0.93 (0.87-0.97) | 0.60 (0.58-0.63) |
| mSOFA >7 | 7 | 703/1181 | 6.87 (2.56-18.40) | <0.001 | 41.46; <0.01 | 0.61 (0.57-0.65) | 0.85 (0.82-0.88) |
| mSOFA >13 | 7 | 257/1181 | 8.30 (3.05-22.58) | <0.001 | 28.69; <0.01 | 0.85 (0.80-0.89) | 0.70 (0.67-0.73) |
| mSOFA >19 | 7 | 34/1181 | 2.40 (0.62-9.22) | NS | 4.87; 0.43 | - | - |
| NHOF ≥2 | 7 | 563/1181 | 6.22 (2.92-13.24) | <0.001 | 31.85; <0.01 | 0.67 (0.63-0.71) | 0.80 (0.77-0.83) |
| NHOF ≥3 | 7 | 300/1181 | 8.16 (3.60-18.51) | <0.001 | 25.06; <0.01 | 0.82 (0.77-0.86) | 0.71 (0.68-0.74) |
| NHOF ≥4 | 7 | 138/1181 | 7.47 (3.62-15.43) | <0.001 | 7.07; 0.21 | 0.89 (0.83-0.94) | 0.64 (0.61-0.67) |
| NHOF ≥5 | 7 | 46/1181 | 9.95 (3.36-29.44) | <0.001 | 4.57; 0.47 | 0.98 (0.88-1.00) | 0.60 (0.57-0.63) |
| Child-Pugh Stage A | 9 | 80/1819 | 0.43 (0.19-0.95) | 0.01 | 11.39; 0.12 | 0.20 (0.12-0.30) | 0.56 (0.54-0.58) |
| Child-Pugh Stage C | 9 | 1370/1819 | 3.95 (2.35-6.66) | <0.001 | 21.82; <0.01 | 0.50 (0.48-0.53) | 0.79 (0.75-0.83) |
| CLIF-SOFA ≥8 | 6 | 839/1039 | 10.23 (3.20-32.71) | <0.001 | 12.39; 0.01 | 0.51 (0.48-0.55) | 0.94 (0.89-0.97) |
| CLIF-SOFA ≥15 | 6 | 315/1039 | 6.56 (2.77-15.53) | <0.001 | 24.71; <0.01 | 0.78 (0.73-0.83) | 0.73 (0.70-0.76) |
| CLIF-SOFA ≥22 | 6 | 34/1039 | 2.89 (1.41-5.91) | 0.003 | 0.83; 0.93 | 0.71 (0.54-0.85) | 0.59 (0.55-0.62) |
| Increased Sofa On Day 3 | 2 | 162/258 | 4.57 (2.33-8.98) | <0.001 | 1.38; 0.24 | 0.71 (0.64-0.78) | 0.65 (0.55-0.74) |
| MELD ≥13 | 8 | 1456/1705 | 4.46 (2.26-8.78) | <0.001 | 13.59; 0.03 | 0.49 (0.47-0.52) | 0.86 (0.81-0.90) |
| MELD ≥18 | 8 | 1197/1705 | 5.70 (3.69-8.81) | <0.001 | 16.37; 0.01 | 0.56 (0.53-0.58) | 0.83 (0.80-0.86) |
| MELD ≥26 | 8 | 807/1705 | 5.10 (3.95-6.59) | <0.001 | 7.62; 0.26 | 0.64 (0.61-0.67) | 0.74 (0.71-0.77) |
| MELD ≥35 | 8 | 394/1705 | 4.74 (3.31-6.80) | <0.001 | 9.75; 0.13 | 0.41 (0.38-0.45) | 0.94 (0.89-0.97) |
| APACHE II ≥20 | 3 | 503/982 | 3.32 (1.44-7.63) | 0.001 | 12.37; <0.01 | 0.60 (0.56-0.64) | 0.73 (0.69-0.77) |
| APACHE II ≥30 | 3 | 166/982 | 5.72 (2.37-13.81) | <0.001 | 5.87; 0.05 | 0.78 (0.72-0.84) | 0.63 (0.56-0.66) |
| APACHE II ≥40 | 3 | 40/982 | 8.46 (2.66-28.81) | <0.001 | 1.12; 0.57 | 0.93 (0.84-1.02) | 0.57 (0.54-0.61) |
| APACHE II ≥50 | 3 | 003/982 | 1.60 (0.31-8.31) | NS | 0.02; 0.98 | - | - |

NS: not significant. PPV : positive predictive value. NPV: negative predictive value. PPV and NPV are given only in case of significant and non heterogeneous results.

GNB: Gram Negative Bacilli. GPB: Gram Positive Bacilli. GPC: Gram positive Cocci. HCC: hepatocellular carcinoma. SIRS: Systemic Inflammatory Response Syndrome. MARS: Molecular Adsorbants Recirculation System. TIPS: Transjugular Intrahepatic Portosystemic Shunt. SOFA: Sequential Organ Failure Assessment. mSOFA: modified SOFA. CLIF-SOFA: modified SOFA according to the Chronic Liver Failure Consortium of the European Association for the Study of the Liver. NHOF: Non-hematologic Organ failure. MELD: Model of End-stage Liver Disease. APACHE: Acute Physiology and Chronic Health Evaluation.

* defined by urine output <0.5mL/kg/h despite adequate fluid resuscitation.

Table S3: Predictors of in-hospital mortality

| **VARIABLES** | **N studies** | **N patients** | **Weight-adjusted OR (95%CI)** | **p** | **Heterogeneity**  **Q ; p** | **PPV (95%CI)** | **NPV (95%CI)** |
| --- | --- | --- | --- | --- | --- | --- | --- |
| **DEMOGRAPHICS** |  |  |  |  |  |  |  |
| Female | 12 | 862/2618 | 0.99 (0.84-1.18) | NS | 7.83; 0.55 | - | - |
| Age>40 | 10 | 2010/2265 | 1.72 (1.31-2.27) | <0.001 | 7.25; 0.51 | 0.55 (0.53-0.57) | 0.56 (0.50-0.63) |
| Age>50 | 10 | 1406/2199 | 1.57 (1.09-2.26) | <0.001 | 24.08; <0.01 | 0.59 (0.56-0.61) | 0.50 (0.47-0.54) |
| Age>60 | 10 | 717/2265 | 1.28 (1.06-1.53) | 0.009 | 5.79; 0.67 | 0.58 (0.54-0.62) | 0.48 (0.45-0.51) |
| Age>70 | 10 |  | 1.13 (0.85-1.51) | NS | 2.58; 0.95 | - | - |
| **DURATION OF ICU STAY** |  |  |  |  |  |  |  |
| ICU Stay<3 days | 10 | 492/2157 | 10.91 (0.71-1.68) | NS | 28.47; <0.01 | - | - |
| ICU Stay>14 days | 10 | 659/2265 | 2.29 (1.39-3.77) | <0.001 | 42.66; <0.01 | 0.75 (0.72-0.79) | 0.48 (0.45-0.50) |
| **ETIOLOGY OF CIRRHOSIS** |  |  |  |  |  | - | - |
| Alcohol | 10 | 1274/2446 | 1.31 (0.70-2.47) | NS | 103.55; <0.01 | - | - |
| Virus | 10 | 445/2241 | 1.17 (0.81-1.64) | NS | 18.41; 0.03 | - | - |
| Metabolic | 8 | 88/1844 | 0.94 (0.62-1.44) | NS | 4.71; 0.69 | - | - |
| **HISTORY OF CIRRHOSIS** |  |  |  |  |  |  |  |
| Past History Of Ascites | 3 | 191/460 | 2.06 (0.43-9.89) | <0.001 | 23.02; <0.01 | 0.69 (0.62-0.75) | 0.60 (0.55-0.66) |
| Past History Of Curable HCC | 2 | 18/277 | 0.51 (0.06-16.66) | NS | 8.36; <0.01 | - | - |
| Past History Of Incurable HCC | 4 | 37/423 | 0.86 (0.27-2.73) | NS | 4.59; 0.20 | - | - |
| Past History Of Variceal Bleeding | 3 | 147/382 | 0.83 (0.42-1.6) | NS | 3.67; 0.19 | - | - |
| **MAIN REASON FOR ADMISSION** |  |  |  |  |  |  |  |
| Variceal Bleeding | 10 | 753/2268 | 0.42 (0.32-0.56) | <0.001 | 13.96; 0.08 | 0.38 (0.35-0.42) | 0.38 (0.36-0.41) |
| Post Operative Management | 8 | 7/1622 | 0.44 (0.15-1.28) | NS | 1.18; 0.99 | - | - |
| Acute Respiratory Failure | 10 | 350/2268 | 1.85 (0.94-3.62) | NS | 42.65; <0.01 | - | - |
| Septic Shock | 8 | 336/1513 | 4.20 (1.88-9.34) | <0.001 | 21.95; <0.01 | 0.80 (0.76-0.84) | 0.52 (0.49-0.55) |
| Acute Renal Failure | 10 | 331/2268 | 1.78 0.78-4.05) | NS | 39.45; <0.01 | - | - |
| Acute-On-Chronic Liver Failure | 5 | 373/811 | 5.50 (1.37-22.02) | <0.001 | 26.07; <0.01 | 0.62 (0.57-0.67) | 0.49 (0.44-0.53) |
| Coma | 10 | 350/2005 | 1.18 (0.61-2.29) | NS | 45.09; <0.01 | - | - |
| **HOSPITALIZATION PRIOR TO ICU ADMISSION** | 5 | 235/554 | 0.92 (0.41-2.03) | NS | 17.46; <0.01 | - | - |
| **CHARACTERISTICS ON ADMISSION** |  |  |  |  |  |  |  |
| Ascites | 5 | 903/1322 | 1.98 (0.57-6.89) | NS | 73.43; <0.01 | - | - |
| Hepatorenal Syndrome | 5 | 64/952 | 1.23 (0.45-3.32) | NS | 5.47; 0.14 | - | - |
| **Parameters of SIRS/infection** |  |  |  |  |  |  |  |
| SIRS according to heart rate | 7 | 790/1704 | 1.07 (0.86-1.33) | NS | 2.37; 0.88 |  |  |
| SIRS according to respiratory rate | 4 | 320/697 | 1.93 (1.37-2.71) | <0.001 | 3.35; 0.34 | 0.65 (0.60-0.70) | 0.51 (0.46-0.56) |
| SIRS according to body temperature | 5 | 500/1271 | 2.52 (1.52-4.19) | <0.001 | 11.78; 0.02 | 0.69 (0.65-0.73) | 0.51 (0.47-0.54) |
| ≥2 criteria for SIRS | 4 | 411/714 | 1.89 (1.25-2.88) | <0.001 | 4.03; 0.25 | 0.70 (0.65-0.74) | 0.53 (0.48-0.59) |
| infection | 6 | 318/1020 | 1.28 (0.42-3.88) | NS | 20.69; <0.01 |  |  |
| Nosocomial Infection | 6 | 100/946 | 3.35 (1.23-9.11) | 0.030 | 7.52; 0.11 | 0.89 (0.81-0.94) | 0.48 (0.44-0.51) |
| Community-Acquired Infection | 5 | 218/811 | 1.55 (0.48-4.94) | NS | 18.83; <0.01 | - | - |
| GNB infection | 5 | 115/811 | 2.39 (1.46-3.91) | <0.001 | 1.59; 0.66 | 0.81 (0.72-0.88) | 0.48 (0.44-0.51) |
| GPB infection | 5 |  | 1.06 (0.39-2.83) | NS | 3.91; 0.27 | - | - |
| GPC infection | 5 | 82/811 | 1.10 (0.59-2.04) | NS | 1.38; 0.35 | - | - |
| Fungal Infection | 5 | 33/811 | 4.52 (1.24-16.47) | 0.004 | 4.03; 0.26 | 0.94 (0.80-0.99) | 0.45 (0.42-0.49) |
| Pneumonia | 7 | 151/1005 | 2.48 (1.44-4.29) | <0.001 | 7.54; 0.18 | 0.77 (0.70-0.84) | 0.48 (0.45-0.52) |
| Urinary Tract Infection | 5 | 37/846 | 1.39 (0.48-4.04) | NS | 4.79; 0.39 | - | - |
| Spontaneous Bacterial Peritonitis | 8 | 131/1264 | 2.11 (1.04-4.29) | 0.003 | 12.92; 0.04 | 0.76 (0.67-0.83) | 0.49 (0.46-0.52) |
| Infection Of Unusual Site | 7 | 42/1005 | 1.35 (0.61-2.99) | NS | 3.41; 0.64 | - | - |
| Cutaneous Infection | 4 | 5/737 | 0.76 (0.20-2.91) | NS | 0.71; 0.86 | - | - |
| Positive Blood Culture | 6 | 142/989 | 2.25 (1.25-4.04) | <0.001 | 7.43; 0.19 | 0.77 (0.69-0.83) | 0.47 (0.44-0.51) |
| Sepsis-Induced Hypotension | 5 | 220/866 | 5.83 (3.38-10.08) | <0.001 | 3.29; 0.35 | 0.84 (0.79-0.89) | 0.52 (0.48-0.56) |
| Sepsis-Induced Refractory Oliguria* | 5 | 187/832 | 7.38 (4.13-13.19) | <0.001 | 3.69; 0.29 | 0.88 (0.82-0.92) | 0.56 (0.53-0.60) |
| Pneumonia Induced Acute Respiratory Failure | 6 | 151/1005 | 6.05 (3.37-10.84) | <0.001 | 2.23; 0.69 | 0.77 (0.70-0.84) | 0.48 (0.45-0.52) |
| **Parameters of renal failure** |  |  |  |  |  |  |  |
| Creatinine ≥ 1.5mg/dL | 11 | 1051/2446 | 3.07 (1.76-5.36) | <0.001 | 78.49; <0.01 | 0.69 (0.66-0.72) | 0.57 (0.55-0.60) |
| Use Of Nephrotoxic Drugs | 3 | 54/360 | 2.15 (0.85-5.41) | NS | 0.11; 0.94 | - | - |
| Oliguria ≤20 ml/h | 7 | 418/1479 | 4.81 (3.43-6.71) | <0.001 | 6.26; 0.28 | 0.82 (0.78-0.85) | 0.55 (0.52-0.58) |
| Creatinine >2.0 mg/dL | 10 | 714/2363 | 3.49 (2.47-4.93) | <0.001 | 18.46; 0.02 | 0.76 (0.73-0.79) | 0.55 (0.53-0.57) |
| **Parameters of respiratory/circulatory failure** |  |  |  |  |  |  |  |
| PaO2<60mmHg | 7 | 122/1728 | 1.88 (1.25-2.83) | 0.002 | 2.41; 0.78 | 0.70 (0.62-0.78) | 0.45 (0.43-0.47) |
| PaCO2>50mmHg | 4 | 87/730 | 1.87 (0.81-5.53) | NS | 5.78; 0.12 | - | - |
| pH<7.3 | 5 | 313/1277 | 2.34 (1.48-3.69) | <0.001 | 7.71; 0.10 | 0.71 (0.66-0.76) | 0.44 (0.41-0.47) |
| PaO2/FiO2<200 | 9 | 636/1922 | 2.70 (1.97-3.71) | <0.001 | 11.42; 0.12 | 0.69 (0.66-0.73) | 0.51 (0.48-0.54) |
| PaO2/FiO2<100 | 9 | 177/1922 | 2.45 (1.69-3.55) | <0.001 | 7.19; 0.41 | 0.75 (0.67-0.81) | 0.46 (0.44-0.49) |
| Mean arterial pressure<65mmHg | 8 | 529/1866 | 2.17 (1.42-3.33) | <0.001 | 15.39; 0.02 | 0.69 (0.65-0.73) | 0.49 (0.46-0.52) |
| **Parameters of neurological failure** |  |  |  |  |  |  |  |
| Hepatic encephalopathy stage 1 | 7 | 432/1479 | 1.21 (0.74-1.93) | NS | 14.20; 0.01 | - | - |
| Hepatic encephalopathy stage 2 | 7 | 240/1479 | 1.38 (0.95-2.01) | NS | 7.05; 0.21 | - | - |
| Hepatic encephalopathy stage 3 | 8 | 342/1920 | 2.64 (1.51-4.59) | <0.001 | 16.53; 0.01 | 0.77 (0.72-0.81) | 0.51 (0.49-0.54) |
| Glasgow Coma Scale ≤7 | 9 | 471/1909 | 2.78 (1.82-4.25) | <0.001 | 16.67; 0.02 | 0.67 (0.63-0.72) | 0.50 (0.48-0.53) |
| Glasgow Coma Scale ≤12 | 9 | 1005/1879 | 2.91 (1.65-5.11) | <0.001 | 44.45; <0.01 | 0.66 (0.63-0.69) | 0.61 (0.58-0.65) |
| **Biochemical parameters** |  |  |  |  |  |  |  |
| Bilirubin>2mg/dL | 10 | 1480/2363 | 3.22 (2.56-4.05) | <0.001 | 9.89; 0.27 | 0.64 (0.62-0.67) | 0.63 (0.59-0.66) |
| Bilirubin>3mg/dL | 10 | 1490/2363 | 3.72 (3.12-4.43) | <0.001 | 5.57; 0.78 | 0.66 (0.63-0.68) | 0.65 (0.62-0.68) |
| Platelet Count <100,000/mm3 | 8 | 1061/2096 | 1.75 (1.12-2.71) | <0.001 | 27.93; <0.01 | 0.60 (0.57-0.63) | 0.54 (0.51-0.57) |
| INR >1.5 | 10 | 1308/2413 | 3.77 (3.04-4.67) | <0.001 | 9.59; 0.29 | 0.68 (0.66-0.71) | 0.60 (0.57-0.63) |
| INR >2.3 | 11 | 737/2410 | 3.89 (2.97-5.11) | <0.001 | 13.53; 014 | 0.74 (0.71-0.77) | 0.55 (0.53-0.57) |
| White Blood Cell count>10,000/mm3 | 8 | 892/1811 | 1.52 (1.25-1.84) | 0.003 | 3.61; 0.73 | 0.61 (0.57-0.64) | 0.49 (0.46-0.52) |
| White Blood Cell count <4,000/mm3 | 7 | 141/1633 | 0.93 (0.46-1.88) | NS | 13.08; 0.02 | - | - |
| CRP>29mg/L | 3 | 265/536 | 0.89 (0.38-2.07) | NS | 6.49; 0.03 | - | - |
| Albumin<28g/L | 7 | 874/1655 | 2.35 (1.18-4.68) | <0.001 | 32.68; <0.01 | 0.62 (0.59-0.65) | 0.54 (0.51-0.58) |
| Natremia<125 mmol/L | 10 | 206/2268 | 1.30 (0.97-1.75) | NS | 6.43; 0.59 | - | - |
| Lactate Upper Normal Value | 7 | 734/1711 | 3.52 (2.50-4.96) | <0.001 | 9.19; 0.10 | 0.71 (0.68-0.74) | 0.56 (0.53-0.59) |
| **THERAPEUTICS** |  |  |  |  |  |  |  |
| Intubation | 10 | 1468/2192 | 5.63 (3.33-9.51) | <0.001 | 43.09; <0.01 | 0.69 (0.67-0.71) | 0.73 (0.70-0.76) |
| Renal Replacement Therapy | 10 | 652/2187 | 4.11 (2.46-6.85) | <0.001 | 29.04; <0.01 | 0.80 (0.77-0.83) | 0.56 (0.53-0.58) |
| MARS | 6 | 48/922 | 1.78 (1.00-3.18) | 0.051 | 1.84; 0.76 | 0.63 (0.47-0.76) | 0.44 (0.41-0.47) |
| TIPS | 3 | 44/728 | 0.25 (0.04-1.41) | 0.036 | 8.14; 0.04 | 0.34 (0.20-0.50) | 0.41 (0.37-0.45) |
| Norephinephrine | 9 | 779/1746 | 8.48 (4.55-15.81) | <0.001 | 28.74; <0.01 | 0.84 (0.81-0.86) | 0.65 (0.62-0.68) |
| Epinephrine | 4 | 66/722 | 7.21 (2.84-18.29) | <0.001 | 1.22; 0.54 | 0.94 (0.85-0.98) | 0.51 (0.47-0.55) |
| Dobutamine | 4 | 29/722 | 9.23 (2.96-28.68) | <0.001 | 1.19; 0.55 | 0.93 (0.77-0.99) | 0.48 (0.45-0.52) |
| Glypressine | 4 | 134/722 | 1.38 (0.84-2.27) | NS | 0.66; 0.71 | - | - |
| Dopamine | 4 | 111/722 | 4.21 (0.48-36.53) | NS | 6.04; 0.05 | - | - |
| Somatostatine | 4 | 210/722 | 0.43 (0.25 -0.75) | <0.001 | 2.87; 0.23 | 0.33 (0.27-0.40) | 0.39 (0.34-0.43) |
| **SCORES** |  |  |  |  |  |  |  |
| SOFA>7 | 9 | 1431/1807 | 9.11 (5.41-15.37) | <0.001 | 15.95; 0.02 | 0.66 (0.63-0.68) | 0.85 (0.81-0.88) |
| SOFA>13 | 9 | 666/1867 | 6.96 (3.84-12.62) | <0.001 | 32.35; <0.01 | 0.86 (0.83-0.88) | 0.59 (0.56-0.62) |
| SOFA >19 | 9 | 163/1782 | 10.31 (2.67-39.79) | <0.001 | 17.67; 0.01 | 0.93 (0.88-0.97) | 0.47 (0.45-0.50) |
| mSOFA>7 | 7 | 697/1172 | 8.06 (4.11-15.81) | <0.001 | 23.52; <0.01 | 0.75 (0.72-0.78) | 0.77 (0.73-0.81) |
| mSOFA>13 | 7 | 255/1172 | 10.13 (3.69-27.79) | <0.001 | 17.78; <0.01 | 0.93 (0.89-0.96) | 0.57 (0.54-0.60) |
| mSOFA>19 | 7 | 40/1180 | 1.67 (0.45-6.19) | NS | 1.38; 0.97 | - | - |
| NHOF≥2 | 7 | 502/1175 | 2.80 (0.77-10.17) | NS | 65.99; <0.01 | - | - |
| NHOF≥3 | 7 | 299/1175 | 8.50 (3.45-20.92) | <0.001 | 20.23; <0.01 | 0.90 (0.86-0.93) | 0.58 (0.55-0.62) |
| NHOF≥4 | 7 | 136/1165 | 9.86 (2.79-34.78) | <0.001 | 9.55; 0.09 | 0.96 (0.91-0.98) | 0.52 (0.49-0.55) |
| NHOF≥5 | 7 | 46/1175 | 8.76 (2.54-30.16) | <0.001 | 4.10; 0.53 | 1.00 (0.92-1.00) | 0.48 (0.45-0.51) |
| Child-Pugh Stage A | 9 | 77/1906 | 0.30 (0.11-0.78) | <0.001 | 15.15; 0.03 | 0.23 (0.14-0.34) | 0.43 (0.41-0.46) |
| Child-Pugh Stage C | 9 | 1409/1891 | 2.35 (0.96-5.70) | NS | 86.41; <0.01 | - | - |
| CLIF-SOFA ≥8 | 6 | 811/1033 | 13.57 (5.63-32.68) | <0.001 | 10.13; 0.04 | 0.64 (0.60-0.67) | 0.90 (0.85-0.94) |
| CLIF-SOFA ≥15 | 6 | 293/1033 | 6.91 (2.58-18.49) | <0.001 | 21.73; <0.01 | 0.88 (0.83-0.91) | 0.59 (0.56-0.63) |
| CLIF-SOFA ≥22 | 6 | 11/1033 | 3.23 (0.77-13.52) | NS | 2.17; 0.70 | - | - |
| increased SOFA on day 3 | 2 | 172/267 | 3.63 (1.31-10.07) | <0.001 | 2.45; 0.11 | - | - |
| MELD ≥13 | 10 | 1957/2318 | 5.01 (3.42-7.34) | <0.001 | 12.61; 0.12 | 0.61 (0.58-0.63) | 0.78 (0.73-0.82) |
| MELD ≥18 | 10 | 1553/2316 | 5.35 (3.41-8.39) | <0.001 | 34.38; <0.01 | 0.68 (0.65-0.70) | 0.72 (0.69-0.75) |
| MELD ≥26 | 10 | 1034/2316 | 4.56 (2.79-7.43) | <0.001 | 47.59; <0.01 | 0.74 (0.71-0.77) | 0.61 (0.58-0.64) |
| MELD ≥35 | 10 | 494/2315 | 3.38 (1.26-9.02) | <0.001 | 96.17; <0.01 | 0.76 (0.72-0.80) | 0.51 (0.49-0.53) |
| APACHE II ≥20 | 4 | 620/1155 | 4.43 (2.57-7.62) | <0.001 | 9.92; 0.01 | 0.75 (0.72-0.78) | 0.61 (0.57-0.65) |
| APACHE II ≥30 | 4 | 200/1155 | 6.41 (3.01-13.67) | <0.001 | 5.49; 0.13 | 0.89 (0.85-0.93) | 0.48 (0.45-0.51) |
| APACHE II ≥40 | 4 | 136/1155 | 10.89 (2.52-47.12) | 0.001 | 0.64; 0.88 | 1.00 (1.00-1.00) | 0.43 (0.40-0.46) |
| APACHE II ≥50 | 4 | 118/1155 | 2.51 (0.43-14.74) | NS | 2.16; 0.53 | - | - |

NS: not significant. PPV: positive predictive value. NPV: negative predictive value. PPV and NPV are given only in case of significant and non heterogeneous results.

GNB: Gram Negative Bacilli. GPB: Gram Positive Bacilli. GPC: Gram positive Cocci. HCC: hepatocellular carcinoma. SIRS: Systemic Inflammatory Response Syndrome. MARS: Molecular Adsorbants Recirculation System. TIPS: Transjugular Intrahepatic Portosystemic Shunt. SOFA: Sequential Organ Failure Assessment. mSOFA: modified SOFA. CLIF-SOFA: modified SOFA according to the Chronic Liver Failure Consortium of the European Association for the Study of the Liver. NHOF: Non-hematologic Organ failure. MELD: Model of End-stage Liver Disease. APACHE: Acute Physiology and Chronic Health Evaluation.

* defined by urine output <0.5mL/kg/h despite adequate fluid resuscitation.

Table S4: Predictors of 6-month mortality in ICU survivors

| **VARIABLES** | **N studies** | **N patients** | **Weight-adjusted OR (95%CI)** | **P** | **Heterogeneity**  **Q ; p** | **PPV (95%CI)** | **NPV (95%CI)** |
| --- | --- | --- | --- | --- | --- | --- | --- |
| **DEMOGRAPHICS** |  |  |  |  |  |  |  |
| Female | 5 | 143/412 | 1.42 (0.92-2.19) | NS | 0.61; 0.96 | - | - |
| Age>40 | 5 | 350/412 | 1.67 (0.78-3.57) | NS | 2.62; 0.62 | - | - |
| Age>50 | 5 | 258/412 | 0.96 (0.61-1.52) | NS | 3.77; 0.43 | - | - |
| Age>60 | 5 | 128/412 | 0.98 (0.53-1.82) | NS | 7.39; 0.116 | - | - |
| **DURATION OF ICU STAY** |  |  |  |  |  |  |  |
| ICU stay<3 days | 5 | 106/387 | 1.36 (0.49-3.77) | NS | 10.93; 0.02 | - | - |
| ICU stay>14 days | 5 | 121/387 | 4.73 (0.35-16.58) | NS | 17.78; <0.01 | - | - |
| **ETIOLOGY OF CIRRHOSIS** |  |  |  |  |  |  |  |
| Alcohol | 5 | 208/387 | 0.66 (0.31-1.44) | NS | 9.19; 0.05 | - | - |
| Virus | 5 | 69/387 | 1.04 (0.39-2.76) | NS | 6.41; 0.17 | - | - |
| Metabolic | 4 | 12/256 | 1.63 (0.50-5.31) | NS | 0.54; 0.91 | - | - |
| **HISTORY OF CIRRHOSIS** |  |  |  |  |  |  |  |
| Past History Of Curable HCC | 2 | 14/118 | 1.06 (0.16-6.79) | NS | 2.15; 0.14 | - | - |
| **MAIN REASON FOR ADMISSION** |  |  |  |  |  |  |  |
| Variceal Bleeding | 5 | 148/387 | 0.71 (0.45-1.12) | NS | 0.22; 0.99 | - | - |
| Post Operative Care | 5 | 0/387 | 1.09 (0.18-6.40) | NS | 0.42; 0.98 | - | - |
| Acute Respiratory Failure | 5 | 62/387 | 1.39 (0.77-2.51) | NS | 1.79; 0.77 | - | - |
| Septic Shock | 4 | 40/268 | 3.95 (1.38-11.30) | 0.010 | 2.50; 0.47 | 0.62 (0.45-0.77) | 0.48 (0.42-0.55) |
| Acute Renal Failure | 5 | 53/357 | 3.29 (1.69-6.40) | <0.001 | 2.05; 0.72 | 0.73 (0.59-0.84) | 0.57 (0.51-0.62) |
| Acute-On-Chronic Liver Failure | 4 | 58/256 | 0.83 (0.36-1.93) | NS | 3.66; 0.30 | - | - |
| Coma | 5 | 72/387 | 0.72 (0.42-1.22) | NS | 2.71; 0.61 | - | - |
| **HOSPITALIZATION PRIOR TO ICU ADMISSION** | 3 | 58/172 | 0.57 (0.29-1.10) | 0.096 | 1.07; 0.58 | - | - |
| **CHARACTERITICS ON ADMISSION** |  |  |  |  |  |  |  |
| Hepatorenal Syndrome | 3 | 18/231 | 4.67 (1.23-17.63) | 0.029 | 0.67; 0.71 | 0.88 (0.65-0.98) | 0.46 (0.40-0.53) |
| **Parameters of SIRS/infection** |  |  |  |  |  |  |  |
| SIRS According To Heart Rate | 3 | 142/278 | 0.85 (0.50-1.46) | NS | 1.31; 0.52 | - | - |
| SIRS According To Respiratory Rate | 3 | 118/278 | 1.04 (0.45-2.40) | NS | 4.95; 0.08 | - | - |
| SIRS According To Body Temperature | 3 | 89/278 | 1.63 (0.87-3.06) | NS | 0.99; 0.61 | - | - |
| ≥2 criteria for SIRS | 3 | 124/249 | 1.07 (0.52-2.22) | NS | 2.63; 0.26 | - | - |
| Infection | 4 | 90/256 | 1.94 (0.76-4.92) | NS | 4.14; 0.24 | - | - |
| Nosocomial Infection | 4 | 34/222 | 2.72 (1.09-6.76) | 0.031 | 0.89; 0.82 | 0.76 (0.58-0.89) | 0.47 (0.41-0.54) |
| Community-Acquired Infection | 4 | 56/256 | 0.92 (0.29-2.91) | NS | 6.71; 0.08 | - | - |
| GNB Infection | 4 | 29/256 | 2.08 (0.87-4.96) | NS | 0.57; 0.91 | - | - |
| GPB Infection | 3 | 4/172 | 2.48 (0.32-19.04) | NS | 0.44; 0.81 | - | - |
| GPC Infection | 4 | 37/256 | 0.83 (0.40-1.72) | NS | 1.21; 0.74 | - | - |
| Fungal Infection | 4 | 4/256 | 2.18 (0.38-12.30) | NS | 0.29; 0.96 | - | - |
| Pneumonia | 4 | 24/260 | 0.85 (0.22-3.25) | NS | 5.76; 0.12 | - | - |
| Urinary Tract Infection | 4 | 23/256 | 2.36 (0.82-6.86) | NS | 2.65; 0.44 | - | - |
| Spontaneous Bacterial Peritonitis | 5 | 43/412 | 1.95 (0.67-5.67) | NS | 6.29; 0.18 | - | - |
| Infection Of Unusual Site | 4 | 12/256 | 0.73 (0.25-2.11) | NS | 0.49; 0.92 | - | - |
| Cutaneous Infection | 3 | 1/179 | 1.23 (0.15-10.26) | NS | 0.31; 0.85 | - | - |
| Positive Blood Culture | 4 | 17/256 | 1.05 (0.38-2.93) | NS | 0.42; 0.93 | - | - |
| Sepsis-Induced Hypotension | 3 | 35/202 | 1.28 (0.38-4.32) | NS | 0.98; 0.61 | - | - |
| Sepsis-Induced Refractory Oliguria* | 2 | 40/177 | 2.06 (0.86-4.90) | NS | 0.72; 0.39 | - | - |
| Pneumonia Induced Acute Respiratory Failure | 3 | 8/203 | 1.57 (0.41-5.92) | NS | 0.46; 0.79 | - | - |
| **Parameters of renal failure** |  |  |  |  |  |  |  |
| Creatinine ≥ 1.5mg/dL | 5 | 130/426 | 1.68 (0.72-3.96) | NS | 10.98; 0.02 | - | - |
| Oliguria ≤20 ml/h | 3 | 36/231 | 2.79 (0.58-13.28) | NS | 3.16; 0.21 | - | - |
| Creatinine>2.0 mg/dL | 4 | 79/333 | 3.01 (0.98-9.34) | NS | 8.33; 0.04 | - | - |
| **Parameters of respiratory & circulatory failure** |  |  |  |  |  |  |  |
| PaO2<60mmHg | 4 | 21/333 | 1.82 (0.71-4.72) | NS | 1.20; 0.75 | - | - |
| PaCO2>50mmHg | 3 | 12/249 | 1.46 (0.45-4.76) | NS | 0.46; 0.79 | - | - |
| pH<7.3 | 3 | 57/249 | 1.19 (0.63-2.26) | NS | 0.01; 0.99 | - | - |
| PaO2/FiO2<200 | 4 | 106/333 | 1.72 (1.03-2.88) | 0.037 | 0.34; 0.95 | - | - |
| Mean Arterial Pressure<65mmHg | 4 | 26/333 | 0.97 (0.42-2.25) | NS | 1.35; 0.71 | - | - |
| **Parameters of neurological failure** |  |  |  |  |  |  |  |
| Hepatic Encephalopathy Stage 1 | 3 | 105/231 | 0.82 (0.37-1.81) | NS | 03.89; 0.14 | - | - |
| Hepatic Encephalopathy Stage 2 | 3 | 53/231 | 0.52 (0.27-1.01) | NS | 0.32; 0.85 | - | - |
| Hepatic Encephalopathy Stage 3 | 3 | 38/231 | 0.91 (0.08-9.54) | NS | 10.29; <0.01 | - | - |
| Glasgow Coma Scale ≤7 | 4 | 122/339 | 0.68 (0.49-0.96) | NS | 9.18; 0.02 | - | - |
| Glasgow Coma Scale ≤12 | 4 | 193/319 | 1.03 (0.20-5.18) | NS | 25.69; <0.01 | - | - |
| **Biochemical parameters** |  |  |  |  |  |  |  |
| Bilirubin>3mg/dL | 4 | 175/333 | 2.14 (1.35-3.39) | 0.001 | 2.49; 0.47 | 0.57 (0.5-0-0.65) | 0.60 (0.52-0.68) |
| Platelet Count <100,000/mm3 | 2 | 164/264 | 1.05 (0.59-1.86) | NS | 0.70; 0.40 | - | - |
| INR >1.5 | 4 | 230/303 | 1.92 (1.16-3.19) | 0.014 | 1.15; 0.76 | 0.53 (0.46-0.60) | 0.61 (0.51-0.70) |
| INR >2.3 | 4 | 113/252 | 3.77 (1.94-7.34) | <0.001 | 4.19; 0.24 | 0.73 (0.64-0.81) | 0.57 (0.48-0.65) |
| White Blood Cell count>10,000/mm3 | 5 | 181/387 | 1.58 (1.03-2.43) | 0.036 | 3.67; 0.45 | 0.52 (0.44-0.59) | 0.58 (0.51-0.65) |
| CRP>29mg/L | 2 | 87/233 | 1.33 (0.61-2.92) | NS | 0.01; 0.91 | - | - |
| Albumin<28g/L | 2 | 158/259 | 1.45 (0.78-2.70) | NS | 0.09; 0.75 | - | - |
| Natremia<125 mmol/L | 5 | 36/389 | 1.93 (0.93-3.93) | NS | 3.03; 0.55 | - | - |
| Lactate Upper Normal Value | 3 | 85/249 | 1.06 (0.47-2.38) | NS | 2.55; 0.28 | - | - |
| **THERAPEUTICS** |  |  |  |  |  |  |  |
| Intubation | 4 | 182/256 | 0.59 (0.16-2.19) | NS | 6.80; 0.07 | - | - |
| Renal Replacement Therapy | 4 | 33/256 | 2.17 (0.92-5.15) | NS | 1.28; 0.73 | - | - |
| MARS | 3 | 7/203 | 2.10 (0.47-9.35) | NS | 0.28; 0.86 | - | - |
| TIPS | 3 | 10/202 | 0.33 (0.10-1.14) | NS | 0.34; 0.84 | - | - |
| Norephinephrine | 4 | 91/256 | 2.07 (1.07-4.00) | 0.029 | 2.08; 0.55 | 0.61 (0.50-0.71) | 0.47 (0.40-0.55) |
| Epinephrine | 3 | 14/231 | 3.78 (0.90-15.87) | NS | 0.31; 0.85 | - | - |
| Dobutamine | 3 | 5/231 | 0.84 (0.10-7.03) | NS | 3.15; 0.21 | - | - |
| Glypressine | 3 | 54/231 | 1.54 (0.69-3.42) | NS | 1.47; 0.47 | - | - |
| Dopamine | 3 | 34/231 | 4.62 (1.64-12.98) | 0.003 | 1.17; 0.55 | 0.88 (0.72-0.96) | 0.49 (0.42-0.56) |
| Somatostatin | 3 | 45/231 | 0.69 (0.35-1.38) | NS | 0.35; 0.83 | - | - |
| **SCORES** |  |  |  |  |  |  |  |
| SOFA>7 | 5 | 266/347 | 3.76 (1.82-7.77) | <0.001 | 5.49; 0.24 | 0.51 (0.44-0.57) | 0.81 (0.71-0.89) |
| SOFA>13 | 5 | 78/377 | 1.79 (0.74-4.32) | NS | 8.03; 0.09 | - | - |
| SOFA >19 | 5 | 7/377 | 2.35 (0.46-12.04) | NS | 1.32; 0.85 | - | - |
| mSOFA>7 | 4 | 184/331 | 5.48 (2.80-10.73) | <0.001 | 4.95; 0.17 | 0.69 (0.61-0.75) | 0.75 (0.67-0.82) |
| mSOFA>13 | 4 | 29/307 | 1.46 (0.46-4.61) | NS | 0.53; 0.91 | - | - |
| mSOFA>19 | 4 | 11/331 | 5.09 (0.87-29.83) | NS | 3.35; 0.34 | - | - |
| NHOF≥2 | 4 | 124/331 | 2.18 (1.18-4.03) | 0.001 | 10.33; 0.24 | 0.62 (0.52-0.70) | 0.58 (0.51-0.65) |
| NHOF≥3 | 4 | 31/331 | 1.15 (0.32-4.11) | NS | 5.98; 0.11 | - | - |
| NHOF≥4 | 4 | 7/324 | 1.56 (0.28-8.67) | NS | 2.28; 0.51 | - | - |
| NHOF≥5 | 4 | 1/331 | 1.27 (0.19-8.19) | NS | 0.20; 0.97 | - | - |
| Child-Pugh Stage C | 4 | 231/324 | 2.43 (1.44-4.10) | <0.001 | 0.53; 0.92 | 0.57 (0.50-0.63) | 0.66 (0.56-0.76) |
| CLIF-SOFA≥8 | 4 | 267/328 | 4.52 (1.91-10.70) | <0.001 | 4.85; 0.18 | 0.55 (0.49-0.61) | 0.77 (0.64-0.86) |
| CLIF-SOFA≥15 | 4 | 56/334 | 1.54 (0.51-4.60) | NS | 7.72; 0.05 | - | - |
| CLIF-SOFA≥22 | 3 | 10/244 | 7.43 (1.18-46.63) | 0.032 | 1.85; 0.39 | 1.00 (0.69-1.00) | 0.55 (0.48-0.62) |
| increased SOFA on day 3 | 2 | 50/112 | 0.80 (0.02-30.92) | 0.200 | 12.42; <0.01 | - | - |
| MELD ≥13 | 4 | 276/318 | 2.36 (1.14-4.91) | NS | 2.48; 0.47 | 0.54 (0.48-0.60) | 0.69 (0.52-0.82) |
| MELD ≥18 | 4 | 187/318 | 3.37 (1.81-6.26) | <0.001 | 3.81; 0.28 | 0.59 (0.52-0.66) | 0.72 (0.63-0.80) |
| MELD ≥26 | 4 | 108/318 | 3.97 (1.92-8.22) | <0.001 | 5.30; 0.15 | 0.75 (0.65-0.82) | 0.60 (0.54-0.67) |
| MELD ≥35 | 4 | 53/318 | 3.39 (1.71-6.74) | <0.001 | 2.51; 0.47 | 0.75 (0.61-0.86) | 0.53 (0.47-0.59) |
| APACHE II ≥20 | 2 | 87/213 | 3.07 (1.64-5.77) | <0.001 | 0.31; 0.57 | 0.68 (0.58-0.78) | 0.67 (0.58-0.75) |
| APACHE II ≥30 | 2 | 21/213 | 6.94 (1.57-37.04) | 0.011 | 0.50; 0.47 | 0.95 (0.76-0.99) | 0.57 (0.50-0.64) |
| APACHE II ≥40 | 2 | 12/213 | 14.58 (1.89-115.76) | 0.010 | 0.28; 0.59 | 1.00 (0.73-1.00) | 0.55 (0.48-0.62) |
| APACHE II ≥50 | 2 | 1/213 | 13.68 (1.74-107.56) | 0.013 | 0.36; 0.54 | 1.00 (0.71-1.00) | 0.55 (0.48-0.62) |
| APACHE II ≥60 | 2 | 1/213 | 13.68 (1.74-107.56) | 0.013 | 0.36; 0.54 | 1.000 (0.71-1.00) | 0.55 (0.40-0.62) |

NS: not significant. PPV: positive predictive value. NPV: negative predictive value. PPV and NPV are given only in case of significant and non heterogeneous results.

GNB: Gram Negative Bacilli. GPB: Gram Positive Bacilli. GPC: Gram positive Cocci. HCC: hepatocellular carcinoma. SIRS: Systemic Inflammatory Response Syndrome. MARS: Molecular Adsorbants Recirculation System. TIPS: Transjugular Intrahepatic Portosystemic Shunt. SOFA: Sequential Organ Failure Assessment. mSOFA: modified SOFA. CLIF-SOFA: modified SOFA according to the Chronic Liver Failure Consortium of the European Association for the Study of the Liver. NHOF: Non-hematologic Organ failure. MELD: Model of End-stage Liver Disease. APACHE: Acute Physiology and Chronic Health Evaluation.

* defined by urine output <0.5mL/kg/h despite adequate fluid resuscitation.

**Figure S1**


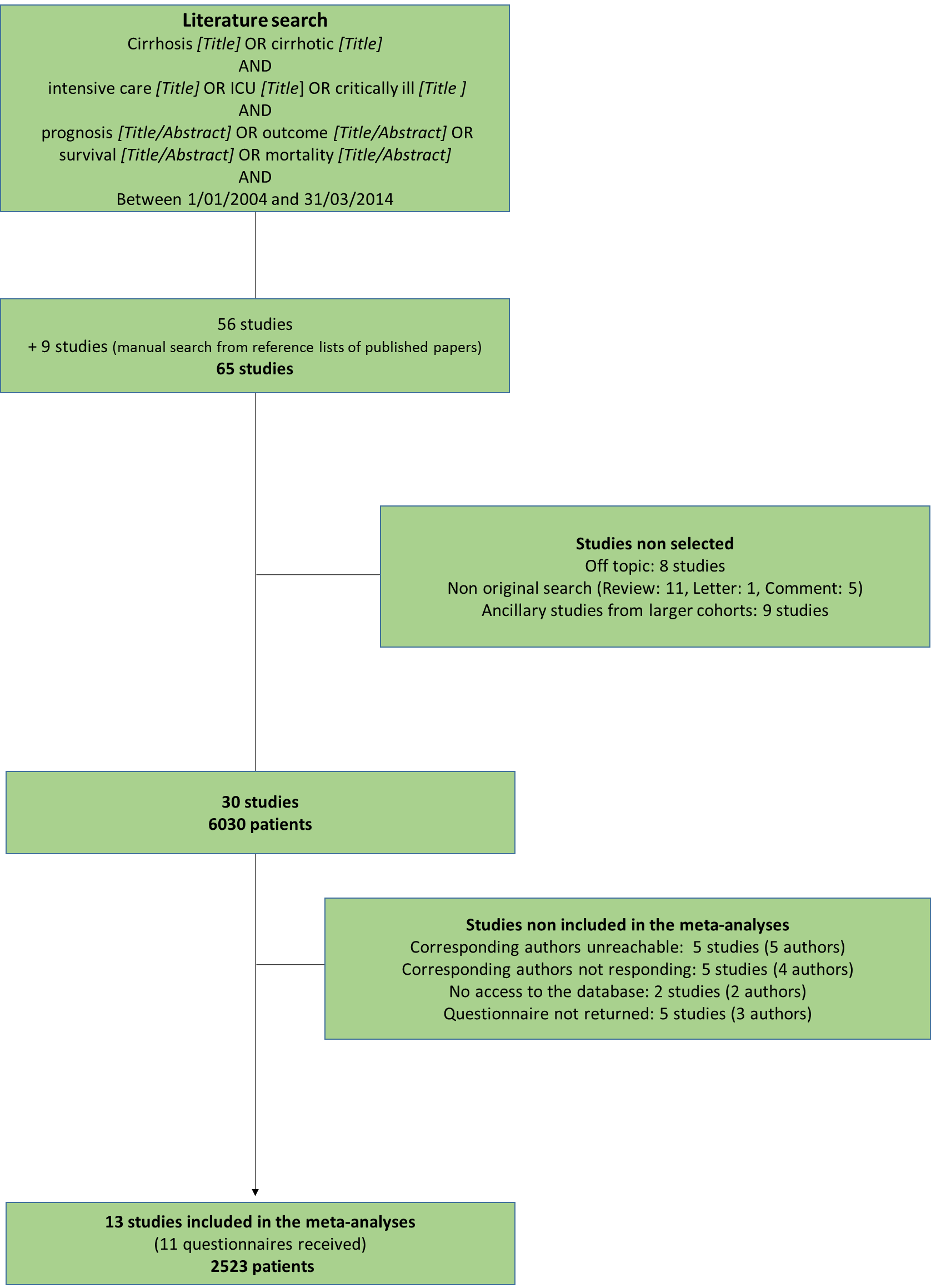


**Legend**: **Flow diagram. Literature search and selection process**
